# Technological Integration in Triage With AI and Machine Learning Compared With Conventional Triage and Narrative Synthesis of Telehealth for Patient Prioritization in Emergency and Acute Care Settings: Systematic Review and Meta-Analysis

**DOI:** 10.2196/93931

**Published:** 2026-10-19

**Authors:** Faii O M Ashour, Waqas Sami, Intima Alrimawi, Moattar Raza Rizvi, Majeda M El-Banna

**Affiliations:** 1 College of Nursing, QU-Health Sector, Qatar University, P.O. Box 2713, Doha, Qatar, +974 4403 7433; 2 Department of Pre-Clinical Affairs, College of Nursing, QU-Health, Qatar University, Doha, Qatar; 3 Berkley School of Nursing, Georgetown University, Washington, DC, United States; 4 Faculty of Allied Health Sciences, Santosh Deemed to be University, Ghaziabad, Delhi, NCR, 201009, India

**Keywords:** artificial intelligence, machine learning, emergency department triage, risk prediction, tele-triage, health equity

## Abstract

**Background:**

Conventional triage relies on clinician judgment and limited information available at first contact. AI and machine learning (ML) may improve early risk stratification, whereas tele-triage and patient-facing tools primarily support remote assessment, access, and care routing.

**Objective:**

This study aimed to evaluate the diagnostic and prognostic performance of AI- and ML-assisted triage systems and summarize evidence on tele-triage, workflow, implementation, economic, and equity outcomes.

**Methods:**

Comparative studies published between January 1, 2010, and December 31, 2025, evaluating AI-, ML-, natural language processing–, telehealth-, or digitally enhanced triage. At first, clinical contacts were included. PubMed/MEDLINE, CINAHL, Web of Science, Scopus, preprint servers, trial registries, and conference proceedings were searched. Screening and data extraction were performed independently in duplicate. Risk of bias was assessed using design-appropriate tools. Clinically comparable area under the receiver operating characteristic curve (AUC) estimates were synthesized using random-effects meta-analysis; heterogeneous diagnostic, operational, implementation, economic, and equity outcomes were summarized narratively. Exploratory scenario modeling projected effects per 10,000 emergency department (ED) visits.

**Results:**

Twenty-seven studies were included, comprising 13 studies in the quantitative evidence stream and 14 in the narrative synthesis. Four adult ED studies evaluating critical care or deterioration outcomes demonstrated high pooled discrimination for AI-assisted triage (AUC 0.874; 95% CI 0.845‐0.898; *I*²=94.4%; 95% prediction interval 0.803‐0.922). Four studies reported directly extractable within-study comparisons favoring AI-assisted models (AUC differences of 0.097, 0.120, 0.108, and 0.065), although these could not be pooled because paired variance estimates were unavailable. Admission/disposition studies reported AI-model AUCs ranging from 0.80 to 0.93 and were summarized descriptively because extreme heterogeneity precluded meaningful pooling. Two pediatric studies (AUCs of 0.85‐0.99) were summarized descriptively. Prediction-model studies were primarily limited by deficiencies in model analysis, calibration reporting, and external validation. Narrative evidence suggested improved front-door operational metrics with tele-triage, including reported reductions of 15%‐40% in time to clinical assessment and 2‐8 percentage points in left-without-being-seen rates, whereas patient-facing symptom checkers generally favored sensitivity over specificity. Exploratory scenario modeling illustrated that, under specified prevalence and threshold assumptions, AI-assisted triage could be associated with 12‐38 fewer missed critical illness cases and 45‐120 additional false-positive escalations per 10,000 ED visits. Economic and equity findings were assumption-dependent projections rather than observed clinical, cost-effectiveness, or equity outcomes.

**Conclusions:**

AI- and ML-assisted triage models demonstrate promising discrimination for adult ED risk stratification; however, AUC-based discrimination does not establish superiority at clinically relevant decision thresholds, improved patient outcomes, or readiness for clinical implementation. Interpretation is limited by substantial heterogeneity, incomplete threshold-level performance and calibration reporting, limited external validation, and sparse pediatric evidence. Prospective multicenter validation, workflow assessment, subgroup calibration and equity auditing, and formal economic evaluation are required before routine implementation.

## Introduction

Triage constitutes the critical first point of contact in patient care, where clinical urgency is assessed and scarce health care resources are prioritized in environments increasingly characterized by crowding, rising demand, and resource constraints [1-3]. Decisions made at this early stage may influence immediate patient safety, downstream clinical outcomes, operational efficiency, and health system performance [1,4]. Undertriage may delay the recognition and treatment of high-acuity illness, whereas overtriage may misallocate limited resources and increase unnecessary escalation [4,5]. Undertriage may delay recognition of serious illness and contribute to preventable deterioration or mortality, whereas overtriage can lead to unnecessary escalation, increased resource consumption, and worsening crowding that adversely affects other patients [6]. As emergency and urgent care systems worldwide face sustained increases in demand alongside workforce constraints, the limitations of traditional triage approaches have become increasingly apparent [2,3].

Conventional triage instruments such as the Emergency Severity Index (ESI), Manchester Triage System (MTS), Canadian Triage and Acuity Scale (CTAS), and Korean Triage and Acuity Scale (KTAS) provide structured frameworks for assigning urgency and prioritizing patients at emergency department (ED) presentation [7-12]. These systems classify urgency using presenting complaints, vital signs, anticipated resource requirements, and clinical assessments at the time of triage. Despite widespread use, their validity and reliability vary across systems, populations, and clinical settings, and interrater agreement is not consistently high [4,5]. Recent evaluations have also shown that undertriage and overtriage remain clinically relevant concerns, particularly when high-acuity illness is not readily apparent from the information available during the initial assessment [4,10].

In response to these challenges, AI and machine learning (ML) have increasingly been evaluated as clinician-facing decision-support approaches for emergency triage [13,14]. AI- and ML-based models can integrate structured electronic health record (EHR) variables, vital signs, previous health care use, and unstructured triage text to estimate clinical risks at or near the time of presentation [15-17]. Natural language processing (NLP) can extract predictive information from triage narratives and nursing assessments that may not be represented in conventional rule-based scales [15,18,19]. Recent reviews and individual comparative studies have reported promising discrimination for critical care, hospitalization, and sepsis-related outcomes, with some studies reporting higher area under the receiver operating characteristic curve (AUC) values than conventional triage approaches [13,14,20]. However, improved discrimination alone does not establish clinical utility or implementation readiness.

Parallel to advances in AI-based decision support, telehealth has expanded the scope of triage beyond physical health care facilities. Tele-triage and virtual front-door models support remote assessment, screening, and patient routing, with potential benefits for access and the management of lower-acuity demand when integrated with in-person emergency care [21,22]. Telehealth use increased substantially during the COVID-19 pandemic; in the United States, telehealth visits during surveillance week 13 of 2020 were 154% higher than during the corresponding week in 2019, while large health systems rapidly expanded video-enabled urgent and nonurgent care [23,24]. During the same period, numerous AI- and ML-based COVID-19 prediction models were rapidly developed, although systematic appraisal identified poor reporting, high risk of bias, and limited external validation [25].

While these experiences demonstrated feasibility and potential benefits, they also highlighted important risks. The rapid development and deployment of inadequately validated prediction models raised concerns regarding risk of bias, overestimated performance, safety, and generalizability [25]. Telehealth access was also uneven, as differences in digital infrastructure, connectivity, digital literacy, and socioeconomic resources could limit participation among underserved populations [26]. Long-term implementation of digital health technologies may be further constrained by technical limitations, workflow disruption, staff workload, training requirements, communication challenges, and insufficient organizational support [27,28]. These concerns underscore the need for rigorous validation, inclusive implementation strategies, and evaluation beyond short-term emergency deployment.

Crucially, the value of technology-enhanced triage depends not only on model discrimination but also on how risk estimates translate into clinical actions. The selection of operating thresholds determines the balance between false negative (FN) safety risks and false positive (FP) escalation burden, with potential implications for resource use and patient flow. Although individual AI-based triage studies have reported favorable discrimination compared with conventional approaches, performance measures such as AUC alone are insufficient to establish clinical utility or readiness for implementation [13,29]. Safe clinical adoption also requires calibration, clinically appropriate thresholds, external validation, workflow and EHR integration, usability, explainability, clinician acceptance, end-user training, regulatory oversight, and postdeployment monitoring [29-32].

Human factors play a central role in determining the effectiveness, appropriate reliance, and sustained use of technology-enhanced triage. Clinician trust in AI-based decision support is influenced by transparency, explainability, clinical reliability, usability, validation, workflow compatibility, training, and the preservation of professional judgment and control [33-35]. Recent user-centered evidence further indicates that interface clarity, usability, workflow fit, customization, and stakeholder involvement are important determinants of the acceptance and integration of clinical decision support systems (CDSSs) [36]. Broader evidence from AI-enabled human-robot interaction in clinical settings suggests that personalization, responsiveness to user expectations, emotional and social interaction, and cultural adaptation may influence patient trust and engagement; however, limited longitudinal evaluation restricts conclusions regarding sustained user engagement [37]. These findings support participatory design, adaptation to local clinical and cultural contexts, and continued assessment of trust, usability, and engagement after implementation. Telehealth services have generally demonstrated favorable patient satisfaction, although satisfaction and acceptance vary according to the delivery modality, population, clinical context, digital access, and measurement method [38].

Tele-triage may redistribute tasks and reduce in-person demands, but it can also introduce communication challenges, altered workload, technical demands, and new training requirements for clinical staff [39-41]. Equity considerations are particularly important in AI- and telehealth-enabled triage. Evidence from health care algorithms indicates that biased outcome definitions, unrepresentative data, and differences in access to care may produce unequal performance or allocation across population groups [42]. Triage-specific studies have also identified differences in urgency assignment according to race, ethnicity, sex, and primary language, highlighting the need to assess whether digital systems reproduce, reduce, or amplify existing disparities [43,44]. Telehealth and other digital interventions may widen existing inequalities when access to devices, connectivity, digital literacy, language support, and culturally appropriate design are not adequately addressed [26,45]. Ethical analyses therefore emphasize transparency, explainability, accountability, privacy protection, and governance throughout algorithmic development and implementation [46]. Equitable adoption requires representative validation data, subgroup-specific performance and calibration assessment, accessible implementation strategies, and continuous postdeployment monitoring.

Economic implications of technology-enhanced triage remain incompletely understood. Implementation may involve costs related to infrastructure, software and EHR integration, staff training, maintenance, data governance, and ongoing performance monitoring; however, these components are not consistently identified or measured across digital health evaluations [47,48]. Broader reviews of digital health interventions suggest potential cost-effectiveness in some settings, but substantial heterogeneity in technologies, comparators, costing methods, and outcome measures limits comparison and transferability across health systems [49]. Economic evidence specific to AI- and telehealth-enhanced triage remains limited, and projected efficiency gains or cost savings should not be interpreted as established economic benefits without prospective evaluation alongside real-world implementation. Transparent reporting using AI-specific economic evaluation guidance is therefore needed [50].

Despite these challenges, AI-, ML-, and telehealth-enabled triage have attracted increasing research and implementation interest. AI- and ML-based systems have primarily been evaluated for clinical risk stratification and outcome prediction, whereas tele-triage interventions mainly support remote assessment, access, patient routing, and workflow management [14,22,31]. However, the evidence remains heterogeneous across populations, clinical settings, intervention functions, comparators, and outcome definitions. AI- and ML-based studies frequently emphasize retrospective discrimination performance, with less consistent reporting of calibration, external validation, patient-centered outcomes, and real-world implementation. Tele-triage studies similarly use diverse interventions and operational outcomes that limit direct comparison [13,14,22]. Consequently, potential improvements in risk identification, patient flow, and access should be regarded as promising rather than established until supported by prospective, multicenter, comparator-based evaluations.

This systematic review and meta-analysis addressed 2 related but distinct questions using a PICOTS (Population, Intervention, Comparator, Outcomes, Timing, and Setting) framework. For the primary diagnostic and prognostic question, the population comprised adults and children undergoing triage at the first point of clinical contact in EDs, urgent or primary care access services, specialty intake settings, or virtual front-door services. The interventions were AI-, ML-, or NLP-assisted triage systems, compared with conventional clinician-led triage, validated triage scales, or clinically relevant reference standards. The primary outcomes were discrimination, calibration, sensitivity, specificity, and patient-important outcomes such as critical care requirement, clinical deterioration, hospital admission, and mortality, assessed during the index acute-care episode or the follow-up period reported by each study. For the secondary questions, tele-triage, patient-facing digital triage tools, computerized decision support systems (DSSs), and workflow-support technologies were evaluated in the same clinical populations and settings. These interventions were compared with usual care, conventional triage, alternative digital approaches, or the comparator reported in each study, with operational, workflow, implementation, usability, economic, and equity outcomes examined. Quantitative synthesis was restricted to clinically comparable diagnostic or prognostic performance measures, whereas heterogeneous secondary outcomes were synthesized narratively. Exploratory scenario modeling was used only to contextualize potential system-level implications.

## Methods

### Study Design, Registration, and Reporting Framework

This systematic review was reported according to the PRISMA (Preferred Reporting Items for Systematic Reviews and Meta-Analyses; Checklist 1) 2020 statement, which provides updated guidance for transparent reporting of systematic reviews and meta-analyses [51]. The quantitative synthesis represented a prediction-model performance meta-analysis based on clinically comparable discrimination (AUC) estimates rather than a conventional diagnostic test accuracy meta-analysis. Relevant items from the PRISMA-DTA (PRISMA extension for Diagnostic Test Accuracy) were applied as a reporting extension where diagnostic accuracy outcomes, comparators, and reference standards were reported [52]. PRISMA-DTA was not used as a separate review design. Reporting of database search strategies followed PRISMA-S (PRISMA literature search extension) recommendations.

The review protocol was prospectively registered in the International Prospective Register of Systematic Reviews (PROSPERO; CRD420251160796) on October 3, 2025, prior to commencement of study screening and data extraction. The review was conducted in accordance with the prespecified protocol, which detailed the objectives, eligibility criteria, search strategy, and analytic plan, including criteria for quantitative synthesis. As this study involved secondary analysis of published literature and did not include the collection of individual-level patient data, institutional ethics approval was not required.

### Eligibility Criteria

Eligible studies evaluated patient-facing or clinician-facing technologies applied at the first point of clinical contact to support triage decision-making or early detection of deterioration, using real-world clinical triage data within routine health care settings. Eligible settings included EDs, urgent and primary care access points, specialty intake clinics, and virtual front-door or telehealth services, with adult and pediatric populations. Interventions of interest included AI- and ML-based DSS, NLP applied to triage-time clinical text or patient-reported symptoms, tele-triage and tele-intake modalities, and EHR-embedded early warning or risk stratification tools designed for use at or immediately after initial assessment. Comparators included conventional triage processes or validated triage scales such as the ESI, MTS, CTAS, and KTAS. Because triage represents an early diagnostic classification task in which patients are assigned to risk or urgency categories at first clinical contact, diagnostic accuracy metrics were prioritized as a methodologically appropriate and commonly reported basis for quantitative synthesis, while operational, workflow, equity, and economic outcomes were synthesized narratively.

Primary outcomes were diagnostic or prognostic performance metrics, including sensitivity, specificity, AUC, and calibration where available. Secondary outcomes included patient-important clinical end points (hospital admission, intensive care unit (ICU) transfer, mortality, and emergency revisit), operational outcomes (time to clinician assessment, ED length of stay (LOS), boarding, and left-without-being-seen), implementation outcomes (acceptability, usability, and fidelity), equity outcomes (subgroup performance by age, sex, race/ethnicity, language, and socioeconomic status), and economic outcomes (implementation and maintenance costs, resource use, and throughput effects). Studies were excluded if they reported only methodological model development without evaluation using real-world clinical triage data and/or without comparison against standard triage practice or an appropriate reference standard, lacked a comparator or reference standard, were single-arm case series with fewer than 10 participants, or were not available in English. For this review, “clinical implementation/evaluation” was defined as evaluation using real-world patient triage encounters (prospective or retrospective), including comparative performance against standard triage practice and/or clinically relevant outcomes; simulation-only studies and purely technical development without clinical evaluation were excluded.

### Information Sources and Search Strategy

Bibliographic databases and supplementary sources were searched for records published from January 1, 2010, to September 7, 2025, and were updated through December 2025. The following databases and platforms were searched: MEDLINE via PubMed; CINAHL via EBSCOhost; Web of Science Core Collection via Clarivate; and Scopus via Elsevier. The search combined controlled vocabulary and free-text terms relating to triage and acute-care access with terms for AI, ML, NLP, clinical decision support (CDS), telehealth, and related digital technologies. Outcome-related concepts included critical care requirements, clinical deterioration, hospital admission, mortality, LOS, revisits, workflow, and implementation outcomes. No study-design filters were applied. Animal-only records were excluded where the platform supported an appropriate human-animal limit. No language restriction was applied during database searching; language eligibility was assessed during full-text screening.

Supplementary searches included medRxiv and arXiv, ClinicalTrials.gov, and the World Health Organization (WHO) International Clinical Trials Registry Platform (ICTRP), relevant conference proceedings, and backward reference-list searching of included studies and relevant reviews. The exact platform, complete line-by-line search strategy, controlled vocabulary, free-text terms, field tags, Boolean and proximity operators, truncation, limits, search date, and number of records retrieved are provided in Multimedia Appendix 1 [53].

### Study Selection and Data Extraction

Records from the original and updated searches were exported separately to EndNote X9 (Clarivate Analytics) for deduplication and subsequently imported into Rayyan (Rayyan Systems, Inc) for screening [54]. Records identified through the updated search were assessed using the same eligibility criteria and duplicate-review procedures as the original search. Titles and abstracts were screened independently by 2 reviewers (FOMA and MME-B), followed by independent full-text assessment of potentially eligible reports. Disagreements were resolved through discussion, with arbitration by a third reviewer when consensus could not be reached. Data extraction was completed independently in duplicate using a piloted standardized form and was based on the complete published reports and available supplementary materials rather than on abstracts alone.

Extracted variables included study design, setting, country, sample size, population characteristics, intervention modality, algorithm family, input data types, comparator characteristics, outcome definitions, effect estimates and associated uncertainty, reference standards, handling of missing data, and implementation characteristics, including integration pathway, training, governance, and usability where reported. When several eligible estimates remained, external-validation estimates were prioritized over internal-validation or development-set estimates, independent test-set results over training-set results, and overall or multicenter estimates over individual-site estimates. The final or principal model identified by the primary study was selected; when no principal model was specified, the eligible model with the highest AUC in the independent validation or test set was selected, provided that it used information available at triage. Where more than one eligible model remained plausible, alternative model selection was examined in sensitivity analysis. Estimates corresponding to the prespecified review outcome and the assessment closest to the triage event were selected, and prespecified or clinically applied thresholds were prioritized over retrospectively optimized thresholds. Information was verified using the full report and supplementary materials, and unavailable details were recorded as “not reported in the full report.” Results from the updated search were incorporated into the PRISMA flow diagram, study-characteristics tables, quantitative and narrative syntheses, and conclusions, as applicable.

### Operationalization of Comparators, Reference Standards, and Evidence-Stream Assignment

Comparators and reference standards were operationalized according to study design. For prediction-model and diagnostic-accuracy studies, comparators included clinician-led triage, usual triage practice, or validated triage scales such as the ESI, MTS, CTAS, and KTAS. Reference standards were the observed clinical end points defined in the primary studies, including critical care intervention or transfer, clinical deterioration, hospital admission, mortality, final diagnosis, or expert-adjudicated urgency. For randomized, nonrandomized, and implementation studies, comparators included usual care, concurrent control groups, preimplementation periods, or alternative digital pathways. A diagnostic reference standard was not required for studies evaluating exclusively operational, workflow, implementation, economic, or equity outcomes. Information was extracted from the main articles and supplementary materials; unclear or unreported details were recorded as such without imputation. Primary-study authors were not contacted for additional information.

Technologies were classified by primary clinical function. AI- and ML-based systems were treated as clinician-facing tools for risk stratification, urgency classification, and outcome prediction. NLP tools were classified by use: triage-text prediction models were included within AI/ML decision support, whereas documentation-focused tools were categorized as workflow support. Tele-triage, patient-facing symptom checkers, computerized DSS, and workflow-support technologies were analyzed as separate functional groups. No pooled analysis combined these distinct intervention categories.

Studies reporting diagnostic or prognostic performance measures were assigned to the quantitative evidence stream. Statistical pooling required at least 3 clinically comparable studies reporting AUC estimates for the same outcome domain with reported or derivable uncertainty. Studies with insufficient uncertainty, incompatible outcomes, or noncomparable performance measures were summarized descriptively rather than pooled. Studies reporting mainly operational, workflow, implementation, safety-proxy, economic, or equity outcomes were assigned to the structured narrative-only stream. Scenario modeling was treated as a secondary analytic subset and was restricted to studies reporting sensitivity and specificity at a defined threshold, confusion-matrix components, or sufficient information to derive these values; studies reporting AUC alone were excluded from scenario modeling.

### Risk of Bias and Applicability Assessment

Risk of bias was assessed using validated tools according to study design. Prediction-model studies were assessed using the Prediction Model Risk of Bias Assessment Tool (PROBAST) [55], diagnostic accuracy studies were assessed using the Quality Assessment of Diagnostic Accuracy Studies-2 (QUADAS-2) [56], nonrandomized comparative studies were assessed using the Risk of Bias in Nonrandomized Studies of Interventions (ROBINS-I) [57], and randomized trials were assessed using the revised Cochrane risk-of-bias tool for randomized trials (RoB 2) [58]. Applicability concerns were recorded alongside risk-of-bias judgments. Assessments were conducted independently by 2 reviewers with consensus resolution.

### Quantitative Synthesis and Statistical Analysis

Quantitative synthesis was performed when at least 3 clinically and methodologically comparable studies reported compatible AUC outcomes for the same domain. The primary pooled effect measure was the AUC, reflecting discriminative performance of AI-assisted triage systems. In the absence of consistently reported paired sensitivity and specificity estimates at comparable thresholds, discrimination-based pooling using logit-transformed AUC values was prespecified as the primary quantitative synthesis approach. Because threshold-specific paired sensitivity and specificity data were unavailable for most studies, clinically comparable AUC estimates were synthesized using random-effects meta-analysis rather than hierarchical summary receiver operating characteristic (HSROC) or bivariate diagnostic accuracy models. Calibration metrics, when reported, were synthesized descriptively but were not pooled quantitatively due to inconsistent reporting across studies.

To stabilize variance for bounded AUC values, each study AUC was transformed to the logit scale using $y_{i}\text{=}log\left(\frac{AUC_{i}}{1\text{-}AUC_{i}}\right)$, a commonly applied transformation for meta-analysis of receiver operating characteristic (ROC)–based summary measures [59]. When 95% CIs were reported, standard errors on the logit scale were derived as $SE_{i}\text{=}\frac{log\left(\frac{AUC_{upper}}{1\text{-}AUC_{upper}}\right)\text{-}log\left(\frac{AUC_{lower}}{1\text{-}AUC_{lower}}\right)}{2\times 1.96},$ which was used as the primary approach for variance estimation; delta-method transformations were applied only when CIs were not available, consistent with established meta-analytic practice for ROC-based measures [59].

Random-effects meta-analysis was conducted using restricted maximum likelihood estimation under the model $y_{i}\text{=}\mu \text{+}u_{i}\text{+}\varepsilon_{i},$ where $u_{i}∼N(0,\tau^{2})$represents between-study variability and $\varepsilon_{i}∼N(0,v_{i})$ represents within-study error, with $v_{i}\text{=}SE_{i}^{2}$, allowing for heterogeneity across studies [60].

Study weights were defined as $w_{i}\text{=}\frac{1}{v_{i}\text{+}\tau^{2}},$ and pooled estimates were back-transformed to the AUC scale using the inverse logit function $AUC\text{=}\frac{e^{\hat{\mu }}}{1\text{+}e^{\hat{\mu }}}.$. Between-study heterogeneity was evaluated using the Cochran Q statistic, the *I*² statistic, and the between-study variance $\tau^{2}$. The *I*² statistic was calculated as $max\left(0,\frac{Q\text{-}(k\text{-}1)}{Q}\right)\times 100\text{\%}$, where *k* is the number of studies [61]. The 95% prediction intervals were computed on the logit scale using a *t* distribution with k-p degrees of freedom, consistent with the Hartung-Knapp framework, as $\hat{\mu }\pm t0.975,k\text{-}p\sqrt{{(\tau }^{2}\text{+}SE_{\hat{\mu }}^{2})}$, and were subsequently back-transformed to the AUC scale to represent the expected range of discriminative performance in future clinical settings [62]. Pooling was restricted to clinically comparable outcome domains to minimize construct heterogeneity across studies. Prespecified sensitivity analyses excluded large national registry studies and restricted analyses to studies using the same comparator triage system. Influence diagnostics were conducted using leave-one-out meta-analysis to evaluate the impact of individual studies on pooled estimates [60]. When fewer than 3 comparable studies were available, outcomes were synthesized descriptively without quantitative pooling. All statistical analyses were performed in R (version 4.5.1; R Core Team) using *metafor* (version 4.8.0) [63].

Across the 4 studies assessing adult emergency department critical care or deterioration outcomes, the pooled AUC was 0.874 (95% CI 0.845-0.898). Between-study heterogeneity was substantial (*I*²=94.4%; τ²=0.022; 95% prediction interval 0.803-0.922).

### Narrative Synthesis

Studies not suitable for pooling were synthesized using a structured narrative approach, grouping evidence by intervention class and outcome domain, with emphasis on operational performance, patient safety proxies, implementation outcomes, equity findings, and economic signals. Narrative synthesis was used to integrate heterogeneous evidence while avoiding inappropriate quantitative aggregation.

### Operational, Economic, and Equity Impact Modeling

In addition to conventional synthesis, scenario-based operational, economic, and equity modeling was conducted to contextualize potential downstream implications of technology-enhanced triage in ED settings. These analyses were exploratory and illustrative rather than formal cost-effectiveness evaluations, given heterogeneity in health care systems and limited standardized reporting of downstream costs and resource use. These modeling analyses were conducted to contextualize diagnostic performance findings in relation to the operational, equity, and economic domains specified in the registered protocol. These analyses represent an exploratory decision-analytic translation of diagnostic performance and do not constitute a formal cost-effectiveness evaluation.

Studies were eligible for modeling if they reported confusion-matrix components at a defined operating threshold, explicit sensitivity and specificity at a specified operating point, or implementation-level performance differences stratified by equity-relevant subgroups, enabling translation of diagnostic performance into operational and equity impact. Studies reporting only discrimination metrics without threshold information were not eligible for modeling but were retained for the AUC meta-analysis.

All modeling outputs were standardized to a hypothetical cohort of 10,000 ED visits. For each eligible study and outcome domain, expected counts of true positives (TPs), FPs, true negatives (TNs), and FNs were computed deterministically from sensitivity, specificity, and outcome prevalence, following standard diagnostic test translation principles [64]. The expected number of outcome-positive encounters was calculated as $N_{positive}\text{=}N\times Prevalence$ and outcome-negative encounters as $N_{\text{negative}}\text{=}N\times \left(1\text{-}Prevalence\right)$, where N=10,000. Expected TPs were calculated as $TP\text{=}Sensitivity\times N_{positive}$, FNs as $FN\text{=}\left(1\text{-}Sensitivity\right)\times N_{positive}$, TNs as $TN\text{=}Specificity\times N_{\text{negative}}$, and FPs as $FP\text{=}\left(1\text{-}Specificity\right)\times N_{\text{negative}}$. When prevalence was reported in the original study, it was used directly; where prevalence was not reported, conservative scenario-based assumptions for critical illness prevalence (typically within a 5%‐10% range, consistent with reported adult emergency department critical care rates) were applied solely for scaling purposes to support standardized translation of diagnostic error counts. While prevalence does not alter relative discrimination between models, it influences absolute projected event counts and cost magnitudes. These prevalence values were therefore used solely for scaling and should not be interpreted as universally applicable baseline risk estimates across health care systems.

Operational burden was approximated through error-count translation, with FPs used as a proxy for additional escalation workload and FNs used as a proxy for missed high-risk cases likely to experience delayed escalation or deterioration, consistent with prior operational modeling in emergency care diagnostics [65]. This approach assumes a linear relationship between error counts and downstream workload burden.

Economic impact was estimated by comparing AI-assisted triage with standard triage under a simplified penalty-based cost framework assuming that the unit cost of a missed critical illness case exceeds the unit cost of a FP escalation. Incremental projected cost was computed as $\Delta Cost\text{=}(\Delta FN\times cost_{FN})\text{+}(\Delta FP\times cost_{FP})$, where $\Delta FN\text{=}FN_{AI}\text{-}FN_{Comp}$ and $\Delta FP\text{=}FP_{AI}\text{-}FP_{Comp}$. Thus, negative values of ΔCost indicate projected cost savings associated with AI-assisted triage. To reflect uncertainty in cost magnitudes, multiple cost scenarios were evaluated, including a base-case scenario and additional scenarios varying the relative penalty assigned to missed cases [66]. This simplified framework translates diagnostic error differences into directional economic implications rather than constituting a full cost-effectiveness evaluation.

A net-benefit analysis was additionally conducted across a plausible range of cost ratios *r*, defined as $r\text{=}cost_{FP}\text{/}cost_{FN}$, and expressed in TP-equivalent units per 10,000 visits as a comparative modeling metric, intended to reflect directional economic impact rather than causal cost-effectiveness, and to allow comparison across studies with differing prevalence and outcome definitions. This approach is conceptually aligned with decision-curve analysis while remaining agnostic to specific willingness-to-pay thresholds [67]. Equity modeling was informed by subgroup-stratified performance differences reported in implementation studies, translating subgroup-specific sensitivity changes into avoided FNs per 10,000 visits within each subgroup and projecting the corresponding change in cost under each cost scenario. These equity estimates were interpreted as directional indicators of potential impact rather than causal estimates, consistent with recommended practice for secondary equity-focused modeling [68]. All meta-analyses, scenario-modeling analyses, and visualization were performed in R version 4.5.1. Meta-analysis was conducted using *metafor* (version 4.8.0), and visualizations were generated using *ggplot2* (version 4.0.3). All translated counts were computed deterministically from reported study metrics and explicitly stated prevalence, threshold, subgroup, and cost assumptions.

### Ethical Considerations

Ethical review and approval were waived for this study, as it involved secondary analysis of published literature and did not involve human participants or identifiable personal data.

## Results

### Search Results

The study-selection process is summarized in the PRISMA 2020 flow diagram (Figure 1). Database searches identified 2766 records, and 110 additional records were identified through supplementary sources, yielding 2876 records before deduplication. After removal of 1613 duplicates, 1263 unique records underwent title and abstract screening. Of these, 1177 records were excluded and 86 full-text articles were assessed for eligibility. Fifty-nine full-text articles were excluded for the reasons presented in Figure 1, leaving 27 studies [47-59,61-74] included in this systematic review. Of these, 13 [18-20,69-71,75-81] were included in the quantitative synthesis (meta-analysis), while 14 [72-74,82-92] were included in the narrative synthesis only.

**Figure 1.**
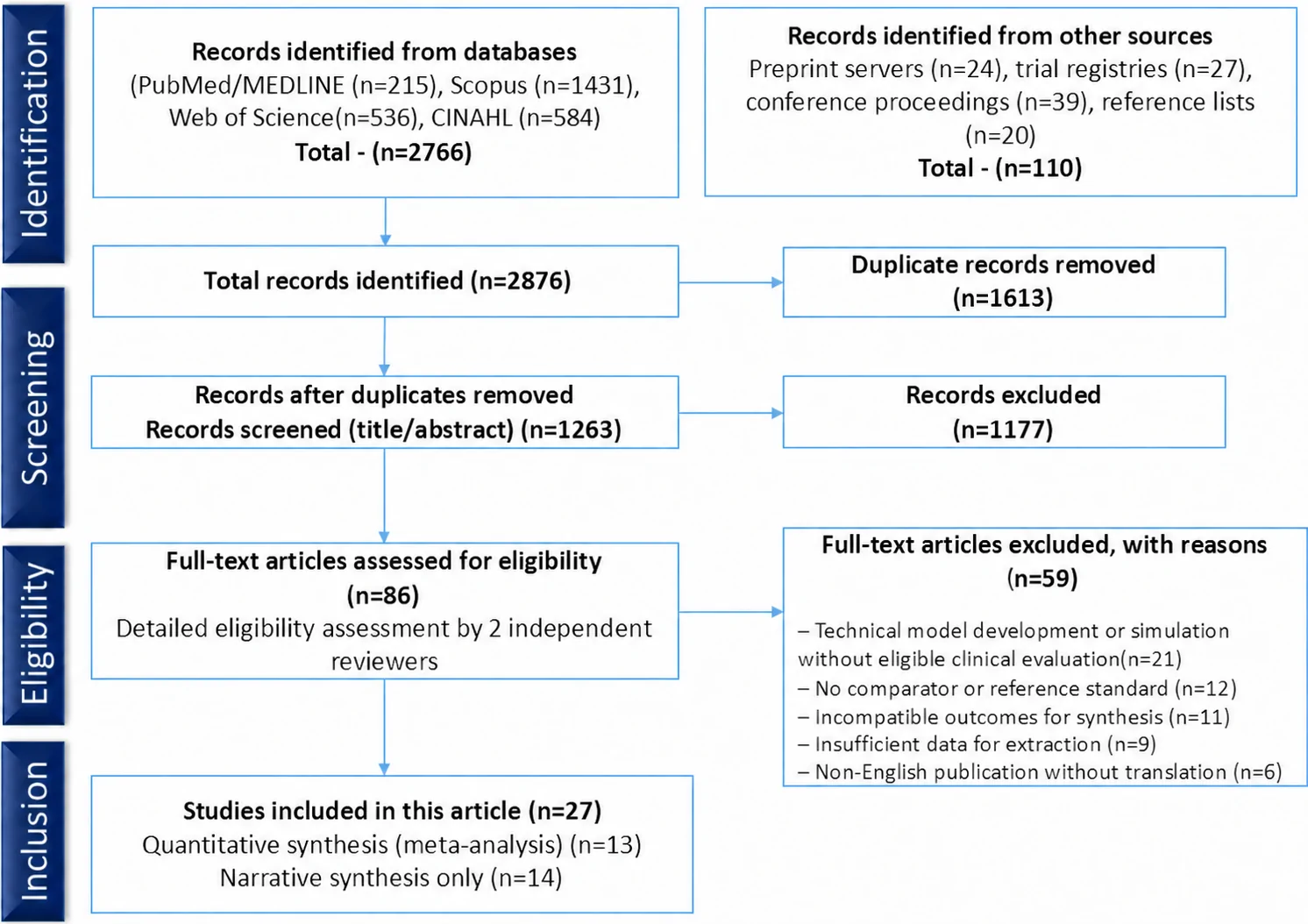
PRISMA (Preferred Reporting Items for Systematic Reviews and Meta-Analyses) 2020 flow diagram showing record identification, duplicate removal, title and abstract screening, full-text eligibility assessment, reasons for exclusion, and final allocation of included studies to the quantitative and structured narrative synthesis streams.

### Study Characteristics

Overall characteristics of the included studies are summarized in Table 1. Of the 27 included studies, 23 were conducted exclusively in ED settings, 2 in urgent or primary care intake settings, and 2 in virtual or telehealth front-door settings. Seventeen studies included adult-only populations, 3 included pediatric-only populations, 2 included mixed adult and pediatric populations, and 5 were classified as other or did not involve patient-level populations. The 13 studies assigned to the quantitative evidence stream comprised 10 evaluations of structured AI- or ML-based triage models, 2 evaluations of AI- or ML-based models incorporating NLP of triage-time free text, and 1 evaluation of a computerized decision-support or electronic triage system (ETS).

**Table 1.** Summary characteristics of included studies (N=27).

Characteristic	Overall (n=27)	Meta-analysis stream (n=13)	Narrative-only stream (n=14)
Population group	Adults: 17; Pediatrics: 3; Mixed: 2; Other/not patient-level: 5	Adults: 10; Pediatrics: 2; Mixed: 1; Other: 0	Adults: 7; Pediatrics: 1; Mixed: 1; Other/not patient-level: 5
Setting	ED^a^: 23; Urgent/primary intake: 2; Virtual/telephone: 2	ED: 13; Urgent/primary intake: 0; Virtual/telephone: 0	ED: 10; Urgent/primary intake: 2; Virtual/telephone: 2
Intervention class	AI/ML^b^ structured: 11; AI/ML+NLP^c^: 3; CDSS^d^/e-triage^e^: 2; Tele-triage/tele-intake: 7; Patient-facing digital triage: 3; Workflow augmentation: 1	AI/ML structured: 10; AI/ML+NLP: 2; CDSS/e-triage: 1	Tele-triage/tele-intake: 7; Patient-facing digital triage: 3; CDSS/e-triage: 1; Workflow augmentation: 1; AI/ML structured: 1; AI/ML+NLP: 1
Comparator (dominant types)	Usual triage/clinician: 8; KTAS^f^: 4; ESI^g^: 3; MTS^h^: 1; Other/unspecified: 11	KTAS: 4; ESI: 2; Usual triage/clinician: 2; Other/unspecified: 5	Usual triage/clinician: 6; ESI: 1; MTS: 1; Other/unspecified: 6

a ED: emergency department.

b ML: machine learning.

c NLP: natural language processing.

d CDSS: clinical decision support system.

e E-triage: electronic triage.

f KTAS: Korean Triage and Acuity Scale.

g ESI: Emergency Severity Index.

h MTS: Manchester Triage System.

The 14 studies assigned to the structured narrative-only stream included 7 evaluations of tele-triage, or tele-intake interventions, 3 evaluations of patient-facing digital triage tools, 1 computerized decision-support or electronic triage study, 1 workflow augmentation study, 1 structured AI- or ML-based study, and 1 AI- or ML-based study incorporating NLP. These studies primarily reported heterogenous operational, safety-proxy, implementation, equity, or process outcomes that were not suitable for quantitative pooling. Detailed characteristics and extractable performance measures for studies assigned to the quantitative evidence stream are provided in Multimedia Appendix 2, whereas characteristics of studies included in the structured narrative-only stream are provided in Multimedia Appendix 3.

### Distribution of Evidence and Outcome Domains

The distribution of 27 included studies according to care setting and primary outcome domain is shown in Figure 2. Most studies evaluated adult ED populations, with the largest group addressing critical care or deterioration prediction, workflow and implementation outcomes, diagnostic accuracy or triage agreement, diagnostic condition-specific prediction, and admission or disposition. Pediatric ED evidence was limited to 2 studies, both addressing critical care or deterioration outcomes. Telephone triage, out-of-hours tele-triage, and primary-care triage were represented by a small number of studies and were synthesized narratively because their outcomes and intervention contexts were not comparable with ED AI- or ML-based triage models.

**Figure 2.**
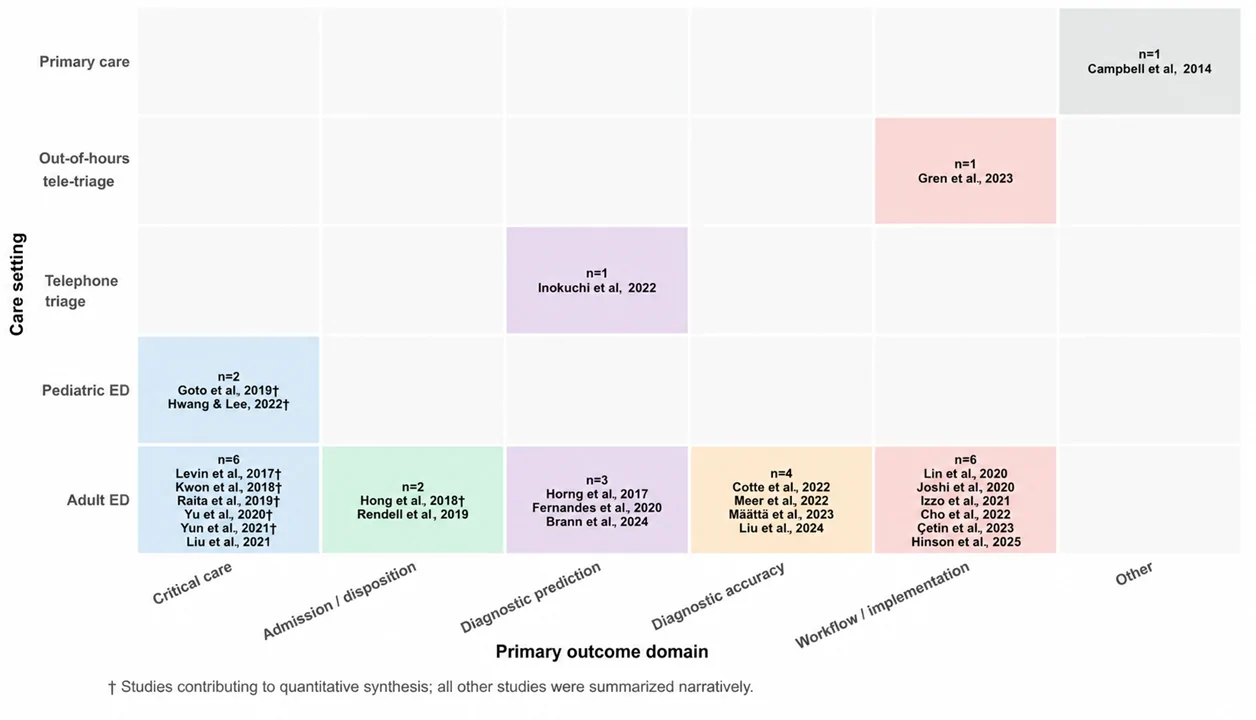
Evidence map of included digital triage studies. Studies are categorized according to care setting and primary outcome domain. Each tile displays the number of studies and corresponding references within each category. The dagger symbol (†) identifies studies assigned to the quantitative evidence stream. Only the 4 adult emergency department critical-care or deterioration studies contributed to the retained pooled analysis; the remaining studies were summarized descriptively or narratively [20,69,75,76]. ED: emergency department.

Studies marked with a dagger in Figure 2 were assigned to the quantitative evidence stream. Of these, 4 adult ED studies reporting critical-care or deterioration outcomes contributed to the retained meta-analysis. The remaining quantitative-stream studies were summarized descriptively because their populations, outcomes, or performance measures were not sufficiently comparable for pooling. Tele-triage, primary care, workflow, implementation, and other heterogeneous studies were retained in the evidence map but were synthesized narratively.

### Quantitative Synthesis: Adult ED Critical Care Outcomes

Four studies involving adult ED populations reported AUC estimates for AI-assisted prediction of critical care requirements or clinical deterioration and met the prespecified criteria for quantitative pooling, although clinical and methodological heterogeneity remained present (Table 2).

**Table 2.** Quantitative synthesis plan and available data for meta-analysis-eligible studies (n=13).

Outcome domain	Metric pooled	Number of eligible studies	Studies contributing (author and year)	Not pooled (reason)
Adult ED^a^ critical care/critical outcome [20,69,75,76]	AUC^b^	4	Yun et al (2021) [69]; Raita et al (2019) [20]; Kwon et al (2018) [75]; Yu et al (2020) [76]	Hinson et al [77] reports implementation sensitivity/specificity changes rather than AUC; Levin et al [70] reports ranges across sites/outcomes not directly poolable; Määttä et al [78] reports urgency agreement/accuracy rather than AUC
Pediatric ED critical care/critical illness [79,80]	Not pooled	2	Goto et al (2019) [79]; Hwang and Lee (2022) [80]	<3 comparable studies (descriptive synthesis)
Admission/hospitalization/disposition [20,75,79-81]	Not pooled	5	Hong et al (2018) [81]; Raita et al (2019) [20]; Goto et al (2019) [79]; Kwon et al (2018) [75]; Hwang and Lee (2022) [80]	Rendell et al [71] admission risk in different context/metrics; Levin et al [70] ranges; heterogeneity in definitions may require secondary sensitivity analysis
Condition-specific detection (sepsis/infection trigger) [18]	Not pooled	2	Horng et al (2017) [18]; Brann et al (2024) [19]	Different reference standards/operating points; insufficient comparable studies for pooling
Urgency classification/triage agreement [78]	Not pooled	1	Määttä et al (2023) [78]	Not an AUC-based discrimination outcome

a ED: emergency department.

b AUC: area under the receiver operating characteristic curve.

Across the 4 studies, individual AUC values for AI-assisted triage ranged from 0.860 to 0.894. Four studies provided directly extractable within-study comparisons with conventional triage. The AUC differences favoring the AI-assisted models were 0.097 (0.894 vs 0.797), 0.120 (0.860 vs 0.740), 0.108 (0.876 vs 0.768), and 0.065 (0.861 vs 0.796), with an unweighted mean difference of 0.098. These contrasts were summarized descriptively and were not pooled because standard errors and covariance estimates for the paired AUCs were unavailable. The Hartung-Knapp-adjusted random-effects meta-analysis of the 4 AI-model AUCs produced a pooled AUC of 0.874 (95% CI 0.845‐0.898), with substantial between-study heterogeneity (*I*²=94.4%; τ²=0.022) and a 95% prediction interval of 0.803‐0.922 (Figure 3). This pooled estimate represents average discrimination across the AI-assisted models and does not constitute a pooled comparative effect against conventional triage.

**Figure 3.**
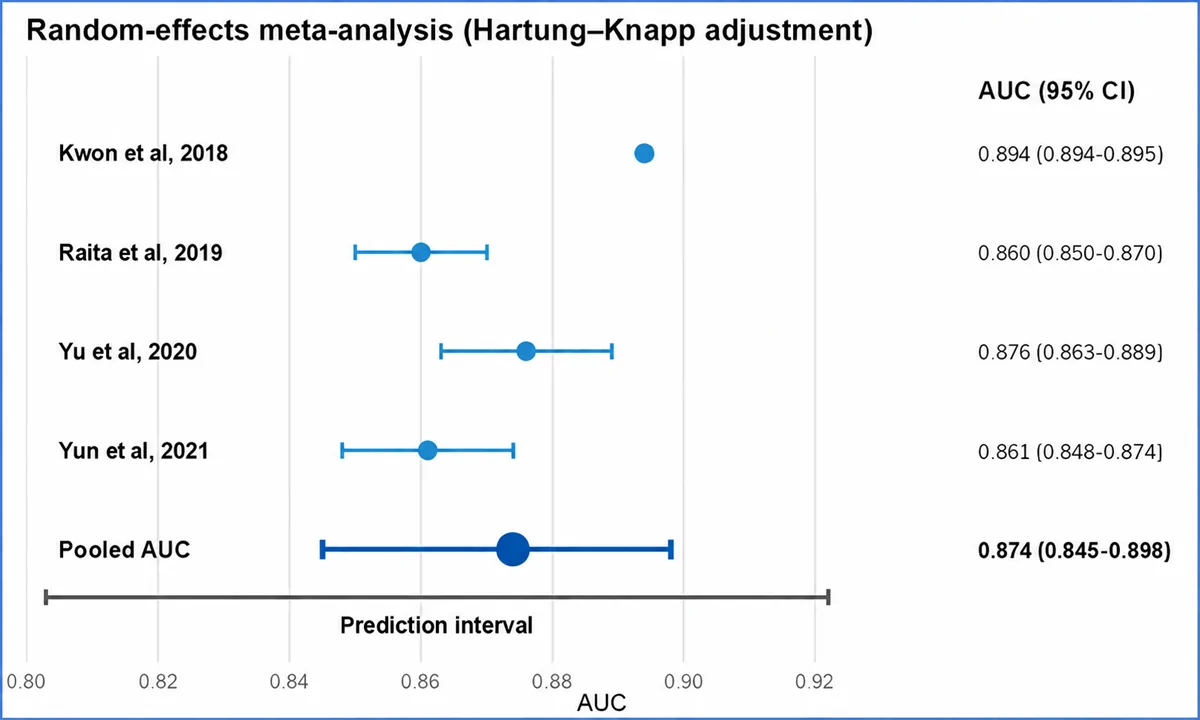
Random-effects meta-analysis (Hartung-Knapp adjustment) of AI-assisted triage discrimination (area under the receiver operating characteristic curve [AUC]) for adult emergency department critical care or deterioration outcomes. Points represent study-specific AUC estimates with 95% CIs. The larger point represents the pooled AUC, and the horizontal line below indicates the prediction interval [20,69,75,76]. AUC: area under the receiver operating characteristic curve.

Substantial between-study heterogeneity was observed after Hartung-Knapp adjustment (*I*²=94.4%), which may reflect differences in model architecture, predictor inputs, validation design, outcome definitions, population characteristics, and comparator triage systems. The 95% prediction interval of 0.803‐0.922 indicates that the expected discrimination of an AI-assisted triage model may vary across future clinical settings. Where conventional triage comparators were reported, study-level AUC estimates for the AI-assisted models were numerically higher; however, no pooled comparative effect was calculated. A sensitivity analysis excluding Kwon et al [75], the largest national registry study, produced a pooled AUC of 0.865 (95% CI 0.842‐0.885), with reduced but still moderate residual heterogeneity (*I*²=47.5%, τ²=0.0025) and a 95% prediction interval of 0.828‐0.895. This indicates that the retained pooled discrimination estimate was not driven solely by the largest registry-scale dataset (Figure 4).

**Figure 4.**
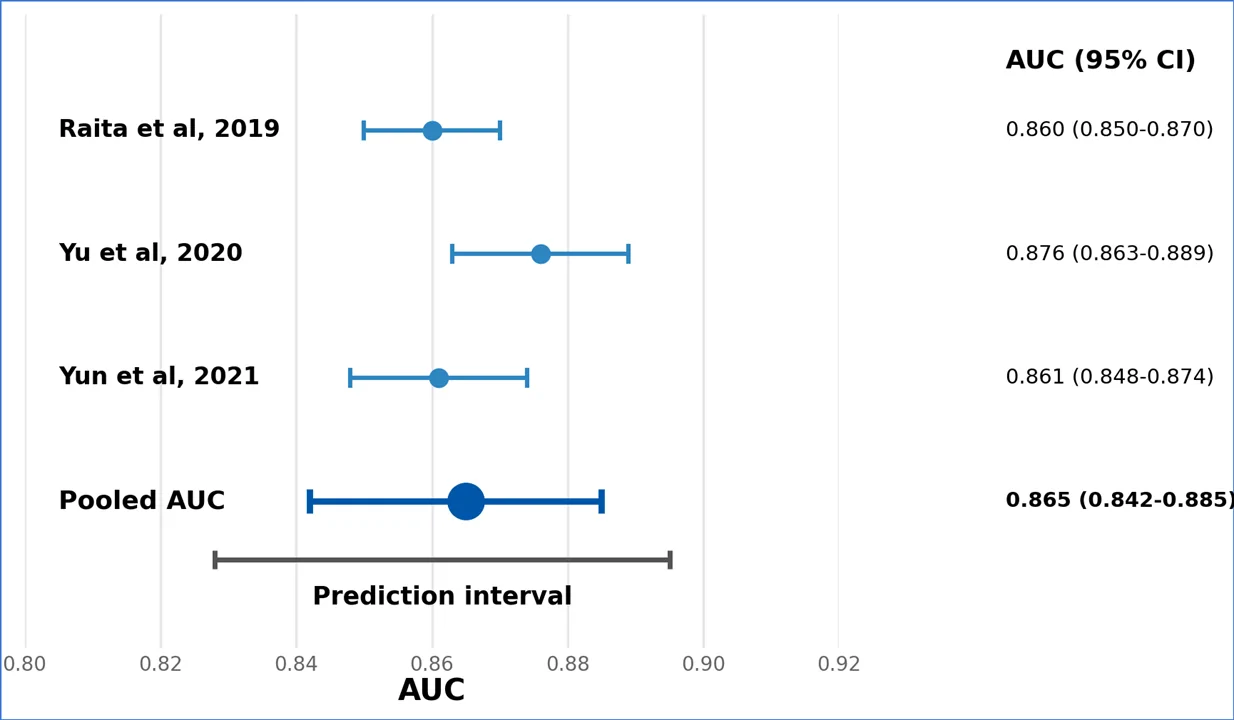
Hartung-Knapp-adjusted sensitivity analysis excluding Kwon et al [75], the largest national registry study. The pooled area under the receiver operating characteristic curve (AUC) remained similar after exclusion, indicating that the overall estimate was not driven solely by the registry dataset. Horizontal bars represent 95% CIs, and the lower line represents the 95% prediction interval [20,69,76]. AUC: area under the receiver operating characteristic curve.

In the leave-one-out influence analysis, sequential exclusion of individual studies produced pooled AUC estimates ranging from 0.865 to 0.878. Although the pooled discrimination estimate remained high after exclusion, removal of Kwon et al [75] reduced heterogeneity from 94.4% to 47.5%, indicating that this large national registry study was an important source of between-study heterogeneity (Figure 5).

**Figure 5.**
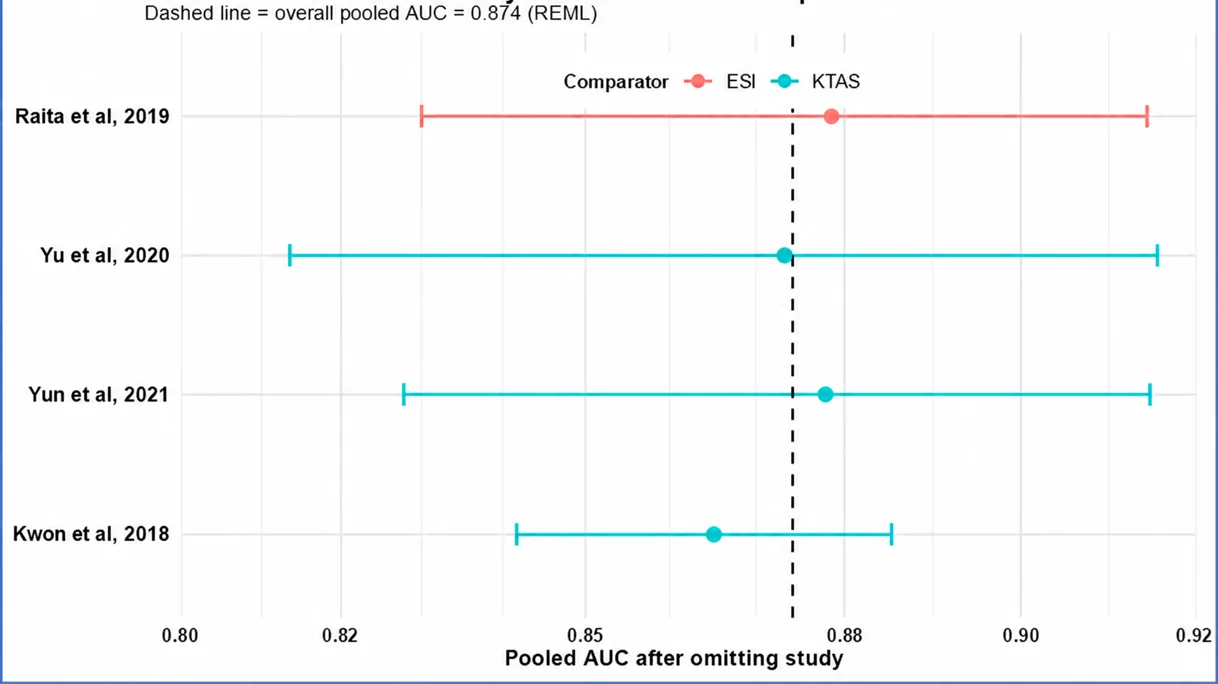
Leave-one-out influence analysis of the random-effects pooled area under the receiver operating characteristic curve (AUC) for AI-assisted prediction of adult emergency department critical care or deterioration outcomes. Each estimate represents the pooled AUC after exclusion of the indicated study, and the dashed vertical line indicates the overall pooled AUC of 0.874 [20,69,75,76]. AUC: area under the receiver operating characteristic curve; ESI: Emergency Severity Index; KTAS: Korean Triage and Acuity Scale.

### Pediatric ED Critical Care Outcomes

Two studies involving pediatric ED populations reported AUC estimates for AI-assisted prediction of critical illness, ranging from 0.85 to 0.99. Where conventional triage comparators were reported, the AI-model AUC estimates were numerically higher at the individual-study level; however, no pooled comparative effect was calculated. Because fewer than 3 studies were available and the outcome definitions differed, quantitative pooling was not undertaken, and the findings were summarized descriptively (Figure 6).

**Figure 6.**
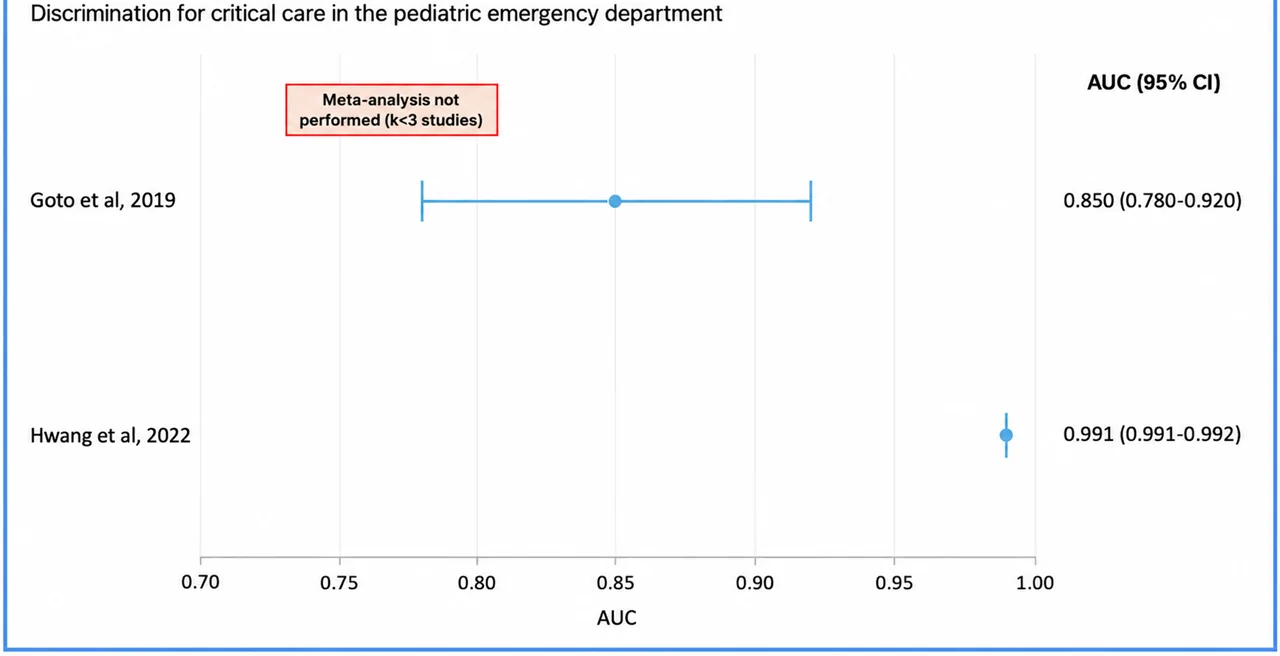
Descriptive area under the receiver operating characteristic curve (AUC) estimates for AI-assisted prediction of critical illness in pediatric emergency department populations. Quantitative pooling was not performed because only 2 clinically heterogeneous studies were available [79,80]. AUC: area under the receiver operating characteristic curve.

The 2 pediatric studies reported high but variable discrimination estimates. However, the small evidence base, differences in outcome definitions, and absence of a pooled estimate limited assessment of the consistency and generalizability of AI-assisted triage performance in pediatric emergency populations.

### Descriptive Synthesis of Hospital Admission and Disposition Outcomes

Five studies reported AUC estimates for admission, hospitalization, or disposition outcomes, including both adult and pediatric ED populations (Table 2). Because these studies combined adult and pediatric populations and used heterogeneous admission-related outcome definitions, this domain was not pooled. Individual AI-model AUCs ranged from 0.80 to 0.93, and where conventional triage or clinician comparators were reported, comparator AUC ranged from 0.72 to 0.80. These findings are therefore presented descriptively (Figure 7).

**Figure 7.**
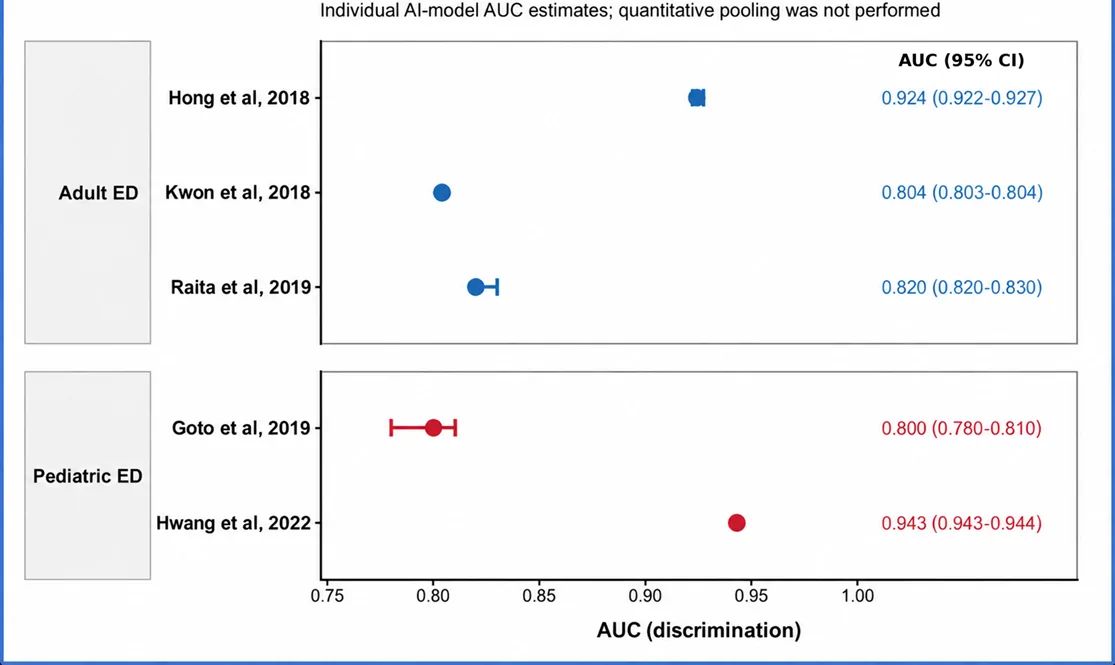
Individual AI-model area under the receiver operating characteristic curve (AUC) estimates for admission/disposition outcomes in adult and pediatric emergency department populations. Findings are presented descriptively because the populations and outcome definitions were not sufficiently comparable for quantitative pooling. Points represent study-specific AUC estimates, and horizontal bars represent 95% CIs [20,75,79-81]. AUC: area under the receiver operating characteristic curve; ED: emergency department.

The admission/disposition studies included both adult and pediatric populations and used heterogeneous outcome definitions, predictor sets, admission thresholds, and health care–system contexts. These findings were therefore summarized descriptively without calculating or interpreting a pooled estimate, heterogeneity statistic, or prediction interval. Individual AI-model AUCs ranged from 0.80 to 0.93, while reported conventional triage or clinician-comparator AUCs ranged from 0.72 to 0.80, summarizing the retained pooled analysis for adult ED critical-care or deterioration outcomes.

### Narrative Synthesis of Studies Not Included in Meta-Analysis

#### Overview

Fourteen studies were included in the structured narrative-only synthesis (Table 3 and Multimedia Appendix 3). To avoid treating technology-enhanced triage as a homogeneous intervention, the evidence was organized according to the primary function of each technology. Tele-triage and tele-intake interventions were synthesized as remote assessment, access management, and patient-routing services. Patient-facing symptom checkers were evaluated separately as tools providing symptom assessment and urgency recommendations. Computerized decision-support and local digital intake systems were synthesized according to their roles in triage-scale alignment and process support, whereas voice- or NLP-enabled systems primarily intended to improve documentation or task completion were classified as workflow-support technologies. AI/ML and NLP-enabled models used for clinical risk prediction were retained within the diagnostic or prognostic evidence stream, unless they lacked comparable performance outcomes. No quantitative analysis combined AI/ML risk-stratification models with tele-triage, patient-facing symptom checkers, or workflow-support systems.

**Table 3.** Characteristics and principal findings of studies included in the narrative synthesis (n=14).

Domain	Intervention category	Principal findings	Included studies
Operational flow and front-door processes [72,73,82-86]	Tele-triage/tele-intake/telemedicine screening	Generally, reported improvements in first-contact routing and throughput measures (eg, time-to-assessment and flow) with variable impact on downstream outcomes depending on local context and implementation.	Campbell et al (2014) [86], Gren et al (2023) [85], Inokuchi et al (2022) [73], Izzo et al (2018) [84], Joshi et al (2020) [82], Lin et al (2020) [72], and Rademacher et al (2019) [83]
Safety proxies and urgency advice [74,87]	Patient-facing symptom checkers/symptom assessment	Typically emphasized conservative urgency advice; usability and safety depend on integration and governance; outcomes are often not comparable for pooling.	Cotte et al (2022) [74], Meer et al (2024) [87]
Documentation/workflow augmentation [88]	Voice AI/documentation support	Evaluated primarily for staff-facing workflow efficiency and documentation support rather than discrimination performance.	Cho et al (2022) [88]
Digital triage DSS^a^ aligned to triage scales [89]	Computerized DSS	Evaluated operational decision support aligned to triage frameworks; outcomes reported as agreement/process measures rather than AUC^b^.	Çetin et al (2023) [89]
Implementation of local digital intake tools [90-92]	Apps/local triage tools	Reported implementation/process outcomes and local performance indicators not harmonized for pooling.	Fernandes et al (2020) [92], Liu et al (2024) [90], Liu et al (2021) [91]

a DSS: decision support system.

b AUC: area under the receiver operating characteristic curve.

#### Tele-Triage and Tele-Intake

Several tele-triage and tele-intake studies reported improvements in front-door operational metrics, although the magnitude and consistency of effects varied across settings and intervention models. Where reported, reductions in time to clinician assessment ranged from 15% to 40%, and reductions in left-without-being-seen rates ranged from 2 to 8 percentage points in high-volume ED settings. Effects on ED LOS were inconsistent, with improvements reported mainly during periods of staffing constraint or increased demand.

#### Patient-Facing Symptom Checkers

Patient-facing digital symptom assessment tools generally produced conservative urgency recommendations. In the included studies reporting threshold-level performance, sensitivity for urgent or emergency conditions exceeded 90%, whereas specificity was lower, resulting in higher rates of overtriage.

#### NLP-Enabled Tools

NLP-enabled tools were classified by function: risk-prediction applications were synthesized with AI/ML decision support, whereas documentation-focused applications were included with workflow-support systems.

#### Workflow Support and Computerized DSSs

The workflow-augmentation study reported improved documentation completeness and reductions of 20%‐35% in triage documentation or task-completion time, but it did not evaluate diagnostic discrimination. The computerized decision-support study reported triage-scale-aligned agreement and process outcomes rather than AUC-based discrimination.

Separately, threshold-level performance data from eligible comparative studies were used in exploratory scenario models standardized to a hypothetical cohort of 10,000 ED visits to estimate projected operational, economic, and equity implications (Figures 8A-8E).

**Figure 8.**
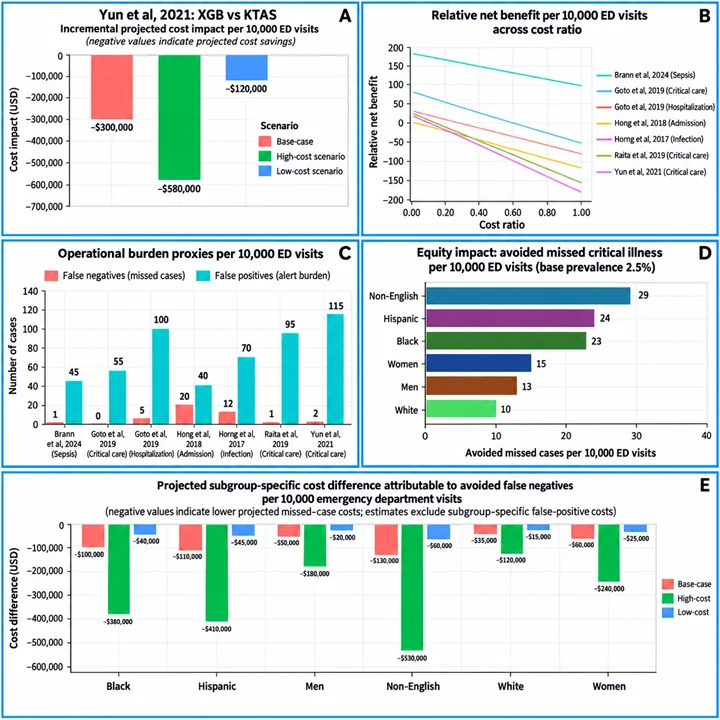
Exploratory scenario-based modeling of possible operational, economic, and equity implications of AI-assisted triage per 10,000 emergency department visits. (A) Incremental projected cost differences under alternative false-negative and false-positive cost assumptions; (B) relative net benefit across specified cost ratios; (C) projected trade-offs between fewer false negatives and additional false-positive escalations; (D) projected avoided false negatives by subgroup using a 2.5% base-case prevalence; and (E) projected subgroup-specific missed-case cost differences attributable to avoided false negatives using a 2.5% base-case prevalence. Negative values indicate lower projected missed-case costs; subgroup-specific false-positive costs were not included. Estimates were derived from study-reported threshold-level performance and explicitly stated prevalence, operating threshold, subgroup, and cost assumptions. These outputs are illustrative and assumption-dependent and should not be interpreted as observed clinical effects, demonstrated cost savings, formal cost-effectiveness estimates, or demonstrated reductions in inequity [18-20,69,79,81]. ED: emergency department; KTAS: Korean Triage and Acuity Scale; XGB: extreme gradient boosting.

#### Operational, Economic, and Equity Impact Modeling

Across the exploratory scenario analyses, and under specified prevalence and threshold assumptions, AI-assisted triage illustrated a potential trade-off of 12‐38 fewer missed critical illness cases and 45‐120 additional FP escalations per 10,000 ED visits, depending on the assumed outcome prevalence and threshold-level sensitivity and specificity differences. Under the specified base-case and higher missed-case penalty assumptions, illustrative cost projections suggested that reduction in FNs could outweigh the estimated cost associated with additional FP escalations. These outputs should be interpreted as directional modeling illustrations, not observed savings or formal cost-effectiveness results.

In exploratory subgroup scenarios, based largely on the limited available subgroup data, including Hinson et al [77], modeling illustrated possible reductions in missed critical illness among some racial or ethnic minority groups and patients with limited English proficiency. The magnitude of the projected reduction varied according to subgroup prevalence, baseline performance, and the assumed change in sensitivity. These findings are assumption-dependent modeling outputs and should not be interpreted as observed reductions in inequity or as evidence that AI-assisted triage will consistently improve equity across settings.

### Risk of Bias Assessment

The methodological quality of the included studies was assessed using risk-of-bias tools appropriate to each study design. Prediction-model studies were evaluated using PROBAST, diagnostic accuracy studies using the QUADAS-2, and nonrandomized intervention studies using the ROBINS-I. The single randomized controlled trial (RCT) was assessed narratively using the RoB 2 framework.

Among the 14 prediction-model studies, PROBAST identified low risk of bias for the participant and predictor domains across all studies, whereas the analysis domain represented the principal methodological limitation. Six studies [19,70,71,76,80,81] (43%) were judged to have a high overall risk of bias, 7 [69,73,75,79,81,91,92] (50%) had an unclear overall risk, and 1 study [91] (7%) was considered to have a low overall risk of bias, largely reflecting limitations in model development, validation, and reporting (Figures 9A-9B).

**Figure 9.**
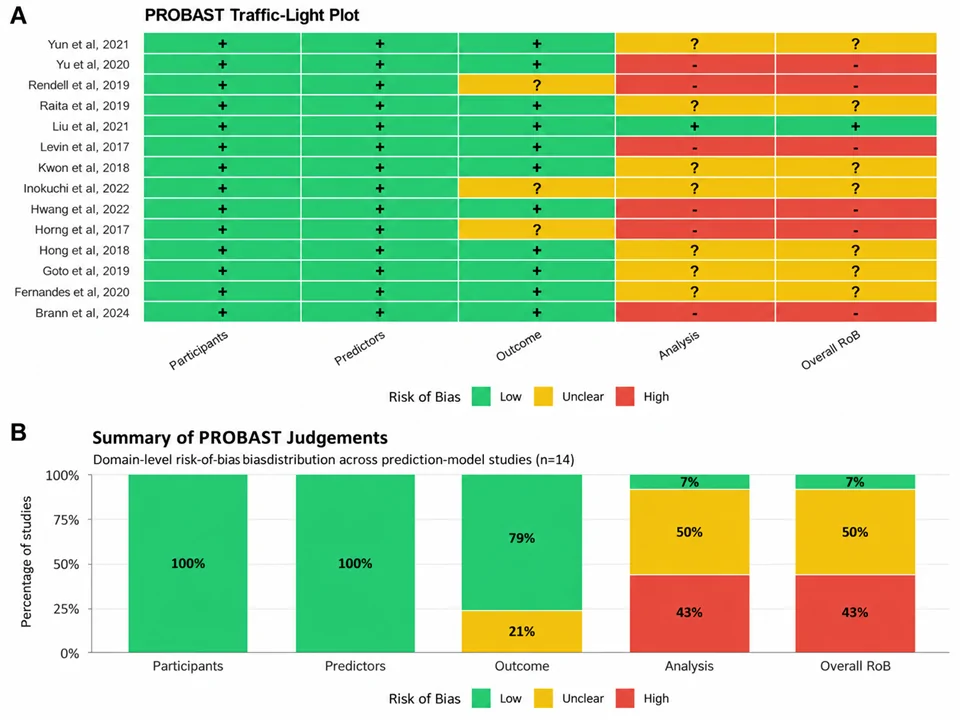
Risk of bias assessment of prediction-model studies (Prediction Model Risk of Bias Assessment Tool [PROBAST]), (A) PROBAST traffic-light plot showing domain-specific risk-of-bias assessments for the 14 prediction-model studies included in the review. (B) Summary of PROBAST risk-of-bias judgments across prediction-model studies. Bars represent the proportion of studies classified as low, unclear, or high risk of bias within each PROBAST domain and for the overall assessment [18-20,69-71,73,75,76,79-81,91,92]. PROBAST: Prediction Model Risk of Bias Assessment Tool; RoB: risk of bias.

The 4 diagnostic accuracy studies [74,78,87,90] demonstrated generally favorable methodological quality. All studies were judged to have a low risk of bias for the index test, reference standard, and flow and timing domains. Concerns were primarily related to patient selection, resulting in 2 studies (50%) being classified as low overall risk of bias, 1 (25%) as unclear, and 1 (25%) as high risk of bias (Figures 10A-10B).

**Figure 10.**
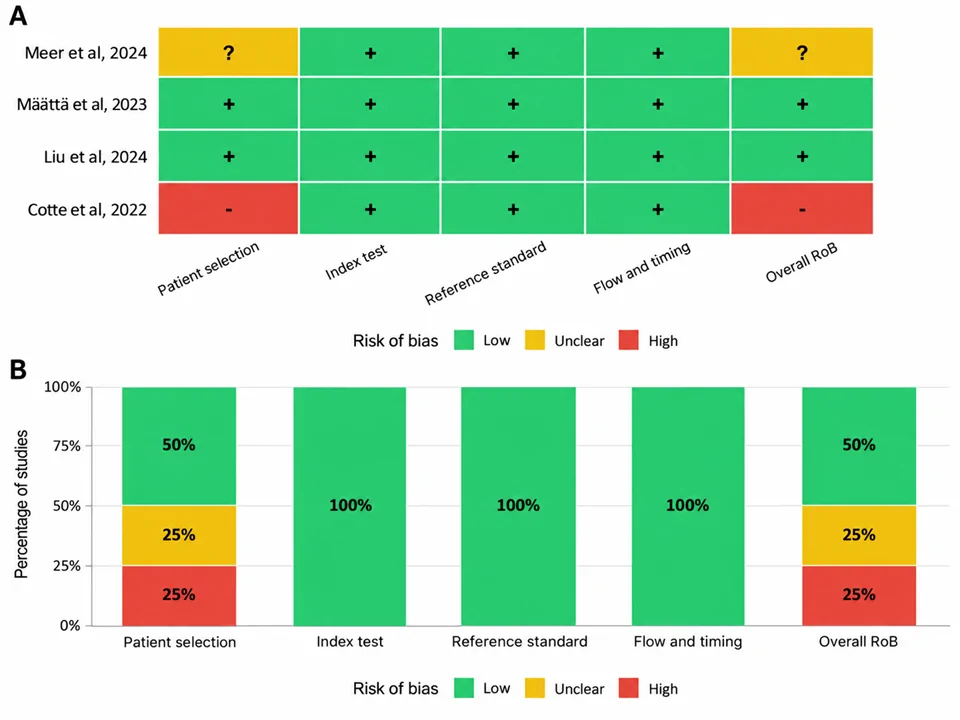
Risk of bias assessment of diagnostic accuracy studies (Quality Assessment of Diagnostic Accuracy Studies-2 [QUADAS-2]), (A) QUADAS-2 traffic-light plot showing domain-specific risk-of-bias assessments for the 4 diagnostic accuracy studies included in the review. (B) Summary of QUADAS-2 risk-of-bias judgments across diagnostic accuracy studies. Bars represent the proportion of studies classified as low, unclear, or high risk of bias within each QUADAS-2 domain and for the overall assessment [74,78,87,90]. RoB: risk of bias; QUADAS-2: Quality Assessment of Diagnostic Accuracy Studies-2.

Risk of bias among 8 nonrandomized intervention studies was assessed using ROBINS-I. Confounding was the principal methodological concern across the observational studies. Overall, 4 studies [83-85,88] (50%) were judged to have a moderate risk of bias and 4 [72,77,82,89] (50%) were judged to have a serious risk of bias, with no study classified as having a critical risk of bias (Figures 11A-11B).

**Figure 11.**
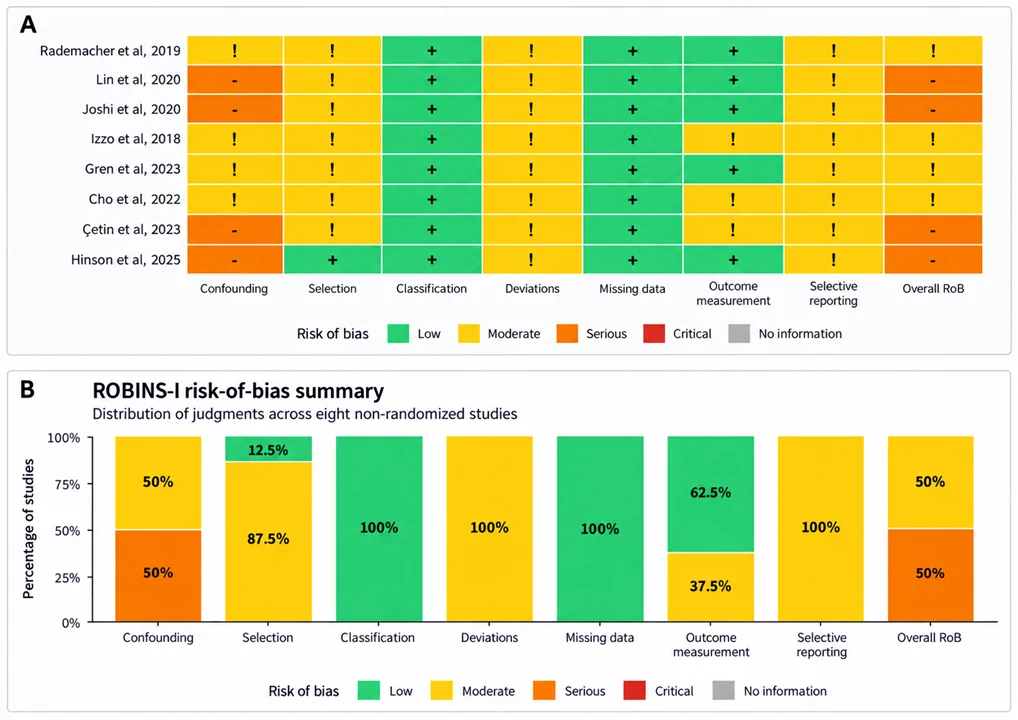
Risk-of-bias assessment of nonrandomized intervention studies using Risk of Bias in Nonrandomized Studies of Interventions (ROBINS-I). Panel A presents domain-specific judgments for the 8 included studies, including Hinson et al [77]. Panel B summarizes the distribution of ROBINS-I judgments across domains and overall assessments. Bars represent the proportions of studies classified as having low, moderate, serious, or critical risk of bias [72,77,82-85,88,89]. RoB: risk of bias.

Hinson et al [77] was retained in the qualitative synthesis because it provided relevant real-world multisite implementation evidence for AI-assisted ED triage. The study was judged to have a serious overall risk of bias, driven primarily by its uncontrolled before-and-after design and the potential for time-varying confounding.

One cluster RCT [86] met the eligibility criteria. As only 1 randomized study was identified, a separate RoB 2 figure was not generated. The trial was judged to have an overall low risk of bias.

## Discussion

### Principal Findings

This systematic review and meta-analysis addressed 2 related objectives: first, to evaluate the diagnostic and prognostic performance of AI- and ML-assisted triage systems; and second, to synthesize evidence on the workflow, implementation, economic, and equity implications of tele-triage and other digital triage technologies. Regarding the first objective, AI-assisted triage systems demonstrated high pooled discriminative performance in adult ED settings, while individual comparative studies reported higher discrimination than conventional triage approaches; however, the quantitative evidence was based on a small number of studies and was characterized by substantial between-study heterogeneity. Pediatric evidence remained limited. Regarding the second objective, tele-triage and patient-facing digital tools were supported mainly by narrative evidence suggesting potential improvements in access, patient routing, and front-door workflow. The scenario-based operational, economic, and equity analyses provided exploratory, assumption-dependent projections rather than observed clinical or system-level effects. Because the available studies primarily reported discrimination measures rather than threshold-specific diagnostic accuracy metrics, the quantitative synthesis focused on pooled AUC estimates rather than hierarchical diagnostic test accuracy modeling.

The high discriminative performance and favorable study-level comparisons observed for AI-assisted triage are consistent with prior work demonstrating the limitations of traditional rule-based triage systems, which rely on a restricted set of snapshot variables and clinician judgment under time pressure [93,94]. ML models, by contrast, can integrate high-dimensional clinical data and capture nonlinear relationships that are difficult to operationalize within conventional triage frameworks [95]. Individual studies included in this review that evaluated electronic or ML-based triage systems against established scales such as the ESI or KTAS generally reported higher risk discrimination for critical care, hospitalization, or deterioration outcomes, supporting the potential of AI as an augmentation to decision support rather than a replacement for clinician judgment [20,70]. However, AUC measures the ability to rank patients by risk rather than the clinical consequences of applying a specific decision threshold. High discrimination therefore does not establish implementation readiness, which requires evaluation of prespecified thresholds, sensitivity-specificity trade-offs, FP escalation burden, FN safety risk, overall and subgroup calibration, workflow integration, equity, and economic feasibility.

Importantly, the findings should be interpreted in the context of substantial clinical and methodological heterogeneity. Differences in outcome definitions, predictor inputs, model architectures, and health care system organization are intrinsic to triage research and have been highlighted in prior evaluations of prediction models in acute care [96-98]. Accordingly, elevated statistical heterogeneity is expected in this domain but materially limits the precision, comparability, and transportability of the pooled estimates. Heterogeneity should therefore not be dismissed as merely contextual variation, even when study-level findings generally point in a similar direction. This reinforces the importance of local calibration, external validation, and contextual assessment, particularly when models are deployed across settings with differing patient populations, clinical thresholds, and operational constraints.

The risk-of-bias findings support cautious interpretation of the observed performance estimates. Prediction-model studies generally demonstrated low risk of bias in participant selection and predictor assessment, but limitations in model analysis, calibration reporting, and external validation were common. Nonrandomized implementation studies were mainly limited by residual confounding associated with their observational designs, whereas diagnostic accuracy studies generally demonstrated better methodological quality. Collectively, these findings indicate that the principal challenge lies not only in achieving high model discrimination but also in ensuring rigorous model development, validation, reporting, and implementation.

Evidence for pediatric triage remains comparatively limited. Although individual pediatric studies reported high discriminative performance for predicting critical illness, the small number of eligible studies and variability in outcome definitions precluded robust quantitative synthesis. Pediatric triage presents distinct challenges related to developmental variability, lower event rates, and increased susceptibility to spectrum effects, all of which may inflate apparent performance in single-center evaluations [99,100]. The current evidence base therefore supports cautious interpretation and underscores the need for prospective, multicenter pediatric evaluations before broader clinical implementation.

The narrative synthesis highlights the complementary but functionally distinct roles of tele-triage, tele-intake, and patient-facing digital triage tools. Unlike AI- and ML-based CDSSs, these interventions primarily support remote assessment, access management, and patient routing. Studies evaluating telephone- and telehealth-based triage reported improvements in access and front-door flow, consistent with prior nurse-led telephone triage and recent ML-enabled tele-triage evaluations [73,101,102]. However, these interventions rarely reported comparator-based diagnostic accuracy metrics and frequently prioritized sensitivity over specificity, limiting their suitability as standalone triage mechanisms in high-acuity emergency settings. Accordingly, the available evidence suggests that tele-triage and digital symptom-assessment tools are best positioned as adjunctive front-door supports that facilitate access and prioritization while preserving clinician oversight.

Beyond diagnostic performance, this review emphasizes the importance of operational, economic, and equity considerations in evaluating triage technologies. Improvements in early risk stratification have the potential to influence downstream outcomes by reducing delays in escalation for high-risk patients and improving resource allocation in crowded EDs [103]. Scenario-based operational and economic modeling provides an exploratory illustration of how changes in sensitivity and specificity might translate into projected differences in missed critical illness, escalation workload, and costs under specified prevalence and cost assumptions. These assumption-dependent projections do not represent observed clinical benefits or formal cost-effectiveness estimates. Rather, they identify potential operational trade-offs that should be evaluated prospectively in real clinical settings before implementation decisions are made.

Equity emerged as a central concern across the included studies. Prior research has demonstrated that algorithmic systems may perpetuate or exacerbate existing disparities if performance is not explicitly evaluated across demographic subgroups [104]. Beyond differences in discrimination, subgroup-specific calibration drift may alter effective clinical decision thresholds, potentially amplifying inequities even when overall AUC remains high. Implementation-focused studies, including multisite evaluations of AI-assisted triage systems, suggest potential reductions in missed high-risk cases among historically underserved populations when subgroup performance is explicitly examined and monitored [77]. Nonetheless, telehealth-based triage interventions also highlighted persistent barriers related to digital literacy, language, connectivity, and insurance status, particularly among underserved and uninsured populations, underscoring the need for inclusive design and governance frameworks that prioritize fairness alongside overall accuracy [29,105,106]. Because subgroup-stratified evidence was limited and the equity modeling relied on available subgroup estimates and stated assumptions, the projected equity effects should be considered exploratory and hypothesis-generating. These findings reinforce the need for subgroup validation, accessible implementation strategies, and continuous postdeployment monitoring in AI- and telehealth-enabled triage systems.

Several limitations of both the included evidence and the review methods should be acknowledged. Most included studies were retrospective and observational, with incomplete reporting of calibration, threshold selection, missing-data handling, and external validation. Limited external validation restricts confidence in transportability across health care systems and underscores the need for prospective, multicenter evaluation before routine deployment. The quantitative synthesis included relatively few studies, and pooling the AI-model AUCs estimated average discrimination rather than a direct within-study comparative effect against conventional triage. The pooled estimate should therefore not be interpreted as definitive evidence that AI-assisted triage is superior to conventional triage. Outcome definitions varied widely, particularly for admission-related end points influenced by local practice patterns. Substantial heterogeneity, especially for admission-related outcomes, further limited the precision, clinical interpretability, and transportability of the findings. Subgroup analyses addressing equity were infrequently reported, and formal economic evaluations were scarce. In addition, the operational, economic, and equity modeling relied on assumptions regarding prevalence, operating thresholds, and cost ratios; these outputs represent illustrative projections rather than observed effects or formal cost-effectiveness estimates. Restriction to English-language studies may also have introduced language bias. Together, these limitations require cautious interpretation and highlight priorities for future research.

### Conclusions

Digital triage technologies should be implemented as function-specific, clinician-supervised tools rather than treated as interchangeable interventions or as replacements for professional judgment. Their adoption should therefore be based not only on discrimination performance but also on demonstrated patient-safety benefits, locally appropriate decision thresholds, calibration, workflow compatibility, interoperability, equity, and economic value in real-world clinical settings.

For health care systems and policymakers, responsible adoption should require prospective external validation, transparent human oversight, subgroup performance auditing, and continuous postdeployment monitoring. Procurement and implementation decisions should distinguish between AI-based risk-prediction systems, tele-triage services, patient-facing symptom checkers, and workflow-support technologies because each serves a different clinical function and is supported by a different type and strength of evidence. Future research should move beyond retrospective model evaluation toward prospective multicenter studies that measure clinical actions, patient outcomes, workload consequences, subgroup effects, and economic outcomes. Such evidence will be essential to determine whether digital triage improves access and prioritization without creating new safety risks, operational burdens, or disparities.

## Supplementary material

10.2196/93931 Multimedia Appendix 1 Full electronic search strategies (PRISMA-S [Preferred Reporting Items for Systematic Reviews and Meta-Analyses extension for Reporting Literature Searches]).

10.2196/93931 Multimedia Appendix 2 Meta-analysis eligible included studies (n=13).

10.2196/93931 Multimedia Appendix 3 Narrative-only included studies (n=14).

10.2196/93931 Checklist 1 PRISMA checklist.

## Abbreviations

AUC: area under the receiver operating characteristic curve
CDS: clinical decision support
CDSS: clinical decision support system
CTAS: Canadian Triage and Acuity Scale
DSS: decision support system
ED: emergency department
EHR: electronic health record
ESI: Emergency Severity Index
ETS: electronic triage system
FN: false negative
FP: false positive
HSROC: hierarchical summary receiver operating characteristic
ICTRP: International Clinical Trials Registry Platform
ICU: intensive care unit
KTAS: Korean Triage and Acuity Scale
LOS: length of stay
ML: machine learning
MTS: Manchester Triage System
NLP: natural language processing
PICOTS: Population, Intervention, Comparator, Outcomes, Timing, and Setting
PRISMA: Preferred Reporting Items for Systematic Reviews and Meta-Analyses
PRISMA-DTA: Preferred Reporting Items for Systematic Reviews and Meta-Analyses extension for Diagnostic Test Accuracy
PRISMA-S: Preferred Reporting Items for Systematic Reviews and Meta-Analyses literature search extension
PROBAST: Prediction model Risk of Bias Assessment Tool
PROSPERO: International Prospective Register of Systematic Reviews
QUADAS-2: Quality Assessment of Diagnostic Accuracy Studies-2
RCT: randomized controlled trial
RoB 2: Revised Cochrane risk-of-bias tool for randomized trials
ROBINS-I: Risk Of Bias in Nonrandomized Studies of Interventions
ROC: receiver operating characteristic
TN: true negative
TP: true positive
WHO: World Health Organization
