# Supplementary material for: Technological Integration in Triage With AI and Machine Learning Compared With Conventional Triage and Narrative Synthesis of Telehealth for Patient Prioritization in Emergency and Acute Care Settings: Systematic Review and Meta-Analysis
Source: J Med Internet Res. 2026 Oct 19;28:e93931. doi: 10.2196/93931 (PMC13679870; doi:10.2196/93931)
Supplement: Supplementary file 1 [file jmir-v28-e93931-s004.pdf]

PRISMA 2020 Checklist

Section and Topic	Item #	Checklist item	Location where item is reported
**TITLE**			
Title	1	Identify the report as a systematic review.	Page 1 – Title page (“Systematic Review and Meta-analysis”)
**ABSTRACT**			
Abstract	2	See the PRISMA 2020 for Abstracts checklist.	Pages 1–2 – Structured Abstract (Background, Objective, Methods, Results, Conclusions)
**INTRODUCTION**			
Rationale	3	Describe the rationale for the review in the context of existing knowledge.	Pages 2–4 – Introduction section
Objectives	4	Provide an explicit statement of the objective(s) or question(s) the review addresses.	Page 2 (Abstract Objective) and Page 4 (Final paragraph of Introduction stating aim)
**METHODS**			
Eligibility criteria	5	Specify the inclusion and exclusion criteria for the review and how studies were grouped for the syntheses.	Page 5 – Section 2.2 “Eligibility Criteria”
Information sources	6	Specify all databases, registers, websites, organisations, reference lists and other sources searched or consulted to identify studies. Specify the date when each source was last searched or consulted.	Pages 5–6 – “Information Sources and Search Strategy” section (search dates reported)
Search strategy	7	Present the full search strategies for all databases, registers and websites, including any filters and limits used.	Supplementary Methods S1 – “Full Electronic Search Strategies (PRISMA-S)”
Selection process	8	Specify the methods used to decide whether a study met the inclusion criteria of the review, including how many reviewers screened each record and each report retrieved, whether they worked independently, and if applicable, details of automation tools used in the process.	Page 6 – “Study Selection and Data Extraction”; independent reviewers; Rayyan software
Data collection process	9	Specify the methods used to collect data from reports, including how many reviewers collected data from each report, whether they worked independently, any processes for obtaining or confirming data from study investigators, and if applicable, details of automation tools used in the process.	Page 6 – “Study Selection and Data Extraction”; duplicate independent extraction; Supplementary Methods S3
Data items	10a	List and define all outcomes for which data were sought. Specify whether all results that were compatible with each outcome domain in each study were sought (e.g. for all measures, time points, analyses), and if not, the methods used to decide which results to collect.	Page 5 – Section 2.2 “Eligibility Criteria” (Primary and Secondary Outcomes)
	10b	List and define all other variables for which data were sought (e.g. participant and intervention characteristics, funding sources). Describe any assumptions made about any missing or unclear information.	Page 6 – “Study Selection and Data Extraction”; Supplementary

Section and Topic	Item #	Checklist item	Location where item is reported
			Methods S3
Study risk of bias assessment	11	Specify the methods used to assess risk of bias in the included studies, including details of the tool(s) used, how many reviewers assessed each study and whether they worked independently, and if applicable, details of automation tools used in the process.	Page 7 – "Risk of Bias and Applicability Assessment"; PROBAST, QUADAS-2, ROBINS-I, RoB2; Supplementary Methods S4
Effect measures	12	Specify for each outcome the effect measure(s) (e.g. risk ratio, mean difference) used in the synthesis or presentation of results.	Pages 7–8 – "Quantitative Synthesis and Statistical Analysis"; AUC primary measure; sensitivity/specificity
Synthesis methods	13a	Describe the processes used to decide which studies were eligible for each synthesis (e.g. tabulating the study intervention characteristics and comparing against the planned groups for each synthesis (item #5)).	Page 7 – Quantitative Synthesis criteria (≥3 comparable studies required for pooling)
	13b	Describe any methods required to prepare the data for presentation or synthesis, such as handling of missing summary statistics, or data conversions.	Pages 7–8 – Logit transformation of AUC; derivation of standard errors
	13c	Describe any methods used to tabulate or visually display results of individual studies and syntheses.	Pages 12–16 – Tables 1–4; Pages 11–22 – Figures 1–10
	13d	Describe any methods used to synthesize results and provide a rationale for the choice(s). If meta-analysis was performed, describe the model(s), method(s) to identify the presence and extent of statistical heterogeneity, and software package(s) used.	Pages 7–8 – Random-effects REML model; I^2, τ^2, Cochran Q; R (metafor package)
	13e	Describe any methods used to explore possible causes of heterogeneity among study results (e.g. subgroup analysis, meta-regression).	Pages 8–9 – Subgroup analysis (adult vs pediatric); heterogeneity statistics
	13f	Describe any sensitivity analyses conducted to assess robustness of the synthesized results.	Pages 8–9 and Page 14 – Registry exclusion analysis; Leave-one-out analysis (Figure 5)
Reporting bias assessment	14	Describe any methods used to assess risk of bias due to missing results in a synthesis (arising from reporting biases).	Page 6 – Preprint and registry searches described; Page 24 – limitation acknowledging no

Section and Topic	Item #	Checklist item	Location where item is reported
			formal funnel plot performed
Certainty assessment	15	Describe any methods used to assess certainty (or confidence) in the body of evidence for an outcome.	Page 24 – No formal GRADE assessment; noted as limitation in Discussion
**RESULTS**			
Study selection	16a	Describe the results of the search and selection process, from the number of records identified in the search to the number of studies included in the review, ideally using a flow diagram.	Pages 10–11 – Results section ("Search Results"); Page 11 – Figure 1 (PRISMA Flow Diagram)
	16b	Cite studies that might appear to meet the inclusion criteria, but which were excluded, and explain why they were excluded.	Page 11 – 48 full-text articles excluded with prespecified reasons (described in Results text; individual citations not listed)
Study characteristics	17	Cite each included study and present its characteristics.	Pages 12–13 – Table 1 (Study Characteristics); Supplementary Tables S1A and S1B
Risk of bias in studies	18	Present assessments of risk of bias for each included study.	Pages 20–22 – Figure 9 (PROBAST traffic-light plot); Figure 10 (QUADAS-2 summary)
Results of individual studies	19	For all outcomes, present, for each study: (a) summary statistics for each group (where appropriate) and (b) an effect estimate and its precision (e.g. confidence/credible interval), ideally using structured tables or plots.	Pages 13–14 – Table 2 (Extractable performance metrics); Pages 13–18 – Forest plots (Figures 3 and 7); Supplementary Table S1A
Results of syntheses	20a	For each synthesis, briefly summarise the characteristics and risk of bias among contributing studies.	Page 16 – Description of adult pooled studies prior to meta-analysis; Risk of bias summary referenced before pooled results
	20b	Present results of all statistical syntheses conducted. If meta-analysis was done, present for each the summary estimate and its precision (e.g. confidence/credible interval) and measures of statistical heterogeneity. If comparing groups, describe the direction of the effect.	Page 13 – Figure 3 (Adult pooled AUC with 95% CI, I^2, τ^2, prediction interval); Page 18 –

PRISMA 2020 Checklist

Section and Topic	Item #	Checklist item	Location where item is reported
			Figure 7 (Pediatric synthesis); Heterogeneity reported in Results text (Pages 13–14)
	20c	Present results of all investigations of possible causes of heterogeneity among study results.	Pages 13–14 – I^2 values and heterogeneity interpretation; Pages 8–9 (Methods referenced in Results); Subgroup discussion in Results narrative
	20d	Present results of all sensitivity analyses conducted to assess the robustness of the synthesized results.	Page 14 – Figure 4 (Registry exclusion sensitivity analysis); Page 14 – Figure 5 (Leave-one-out influence analysis)
Reporting biases	21	Present assessments of risk of bias due to missing results (arising from reporting biases) for each synthesis assessed.	Not formally assessed (no funnel plot presented); acknowledged as limitation on Page 24
Certainty of evidence	22	Present assessments of certainty (or confidence) in the body of evidence for each outcome assessed.	No formal GRADE assessment presented; noted as limitation on Page 24
**DISCUSSION**			
Discussion	23a	Provide a general interpretation of the results in the context of other evidence.	Pages 22–24 – Discussion section (interpretation of pooled AUC findings; comparison with prior ML/triage literature; contextualization of adult vs pediatric findings)
	23b	Discuss any limitations of the evidence included in the review.	Page 24 – Discussion (“Limitations” paragraph addressing heterogeneity, retrospective designs, risk of bias, generalizability concerns)
	23c	Discuss any limitations of the review processes used.	Page 24 – Discussion

PRISMA 2020 Checklist

Section and Topic	Item #	Checklist item	Location where item is reported
			(acknowledgement of no formal reporting bias assessment and no GRADE certainty evaluation; potential publication bias)
	23d	Discuss implications of the results for practice, policy, and future research.	Pages 24–25 – Discussion and Conclusions (implications for ED workflow integration, prospective validation needs, governance, and equity considerations)
**OTHER INFORMATION**			
Registration and protocol	24a	Provide registration information for the review, including register name and registration number, or state that the review was not registered.	Page 4 – Methods section ("Study Design") – PROSPERO registration: CRD420251160796
	24b	Indicate where the review protocol can be accessed, or state that a protocol was not prepared.	Page 4 – Methods section – PROSPERO record referenced as publicly available protocol
	24c	Describe and explain any amendments to information provided at registration or in the protocol.	No amendments reported – not explicitly stated in manuscript
Support	25	Describe sources of financial or non-financial support for the review, and the role of the funders or sponsors in the review.	Page 25 – Funding statement
Competing interests	26	Declare any competing interests of review authors.	Page 25 – Conflicts of Interest section
Availability of data, code and other materials	27	Report which of the following are publicly available and where they can be found: template data collection forms; data extracted from included studies; data used for all analyses; analytic code; any other materials used in the review.	Page 25 – Data Availability Statement; Supplementary Materials referenced (Tables S1A, S1B; Search Strategy)

From: Page MJ, McKenzie JE, Bossuyt PM, Boutron I, Hoffmann TC, Mulrow CD, et al. The PRISMA 2020 statement: an updated guideline for reporting systematic reviews. BMJ 2021;372:n71. doi: 10.1136/bmj.n71. This work is licensed under CC BY 4.0. To view a copy of this license, visit https://creativecommons.org/licenses/by/4.0/
