# Supplementary material for: Technological Integration in Triage With AI and Machine Learning Compared With Conventional Triage and Narrative Synthesis of Telehealth for Patient Prioritization in Emergency and Acute Care Settings: Systematic Review and Meta-Analysis
Source: J Med Internet Res. 2026 Oct 19;28:e93931. doi: 10.2196/93931 (PMC13679870; doi:10.2196/93931)
Supplement: Supplementary file 2 [file jmir-v28-e93931-s001.docx]

**Supplementary Table Sx. Complete updated database search strategies**

**Search period:** 1 January 2010 to 24 December 2026
**Language restriction:** None during searching
**Study-design filter:** None
**Search update date:** 25 June 2026

**1. MEDLINE via PubMed**

**Platform:** PubMed
**Fields:** MeSH and title/abstract
**Limits:** Publication date; animal-only records excluded

(

"Triage"[Mesh]

OR triage[tiab]

OR triaging[tiab]

OR "emergency department triage"[tiab]

OR "emergency triage"[tiab]

OR "urgent care triage"[tiab]

OR "acute care triage"[tiab]

OR "telephone triage"[tiab]

OR teletriage[tiab]

OR "tele-triage"[tiab]

OR "video triage"[tiab]

OR "remote triage"[tiab]

OR "virtual triage"[tiab]

OR "digital triage"[tiab]

OR "electronic triage"[tiab]

OR "e-triage"[tiab]

OR "patient prioritization"[tiab]

OR "patient prioritisation"[tiab]

OR "clinical prioritization"[tiab]

OR "clinical prioritisation"[tiab]

OR "virtual front door"[tiab]

OR "digital front door"[tiab]

)

AND

(

"Artificial Intelligence"[Mesh]

OR "Machine Learning"[Mesh]

OR "Natural Language Processing"[Mesh]

OR "Telemedicine"[Mesh]

OR "Remote Consultation"[Mesh]

OR "Decision Support Systems, Clinical"[Mesh]

OR "artificial intelligence"[tiab]

OR "machine learning"[tiab]

OR "deep learning"[tiab]

OR "neural network"[tiab]

OR "neural networks"[tiab]

OR "random forest"[tiab]

OR "random forests"[tiab]

OR "gradient boosting"[tiab]

OR "gradient-boosted"[tiab]

OR XGBoost[tiab]

OR "support vector machine"[tiab]

OR "support vector machines"[tiab]

OR transformer[tiab]

OR transformers[tiab]

OR "large language model"[tiab]

OR "large language models"[tiab]

OR "natural language processing"[tiab]

OR NLP[tiab]

OR "predictive model"[tiab]

OR "predictive models"[tiab]

OR "prediction model"[tiab]

OR "prediction models"[tiab]

OR "risk prediction"[tiab]

OR "clinical decision support"[tiab]

OR "decision support system"[tiab]

OR "decision support systems"[tiab]

OR computerized[tiab]

OR computerised[tiab]

OR automated[tiab]

OR automation[tiab]

OR telehealth[tiab]

OR telemedicine[tiab]

OR "remote consultation"[tiab]

OR "remote assessment"[tiab]

OR "digital health"[tiab]

OR ehealth[tiab]

OR "e-health"[tiab]

OR mhealth[tiab]

OR "m-health"[tiab]

OR "symptom checker"[tiab]

OR "symptom checkers"[tiab]

OR "symptom assessment tool"[tiab]

OR "symptom assessment tools"[tiab]

)

AND

(

"2010/01/01"[Date - Publication] :

"2026/06/24"[Date - Publication]

)

NOT

(

animals[mh] NOT humans[mh]

)

Record the exact number retrieved on the search date.

**2. CINAHL via EBSCOhost**

**Platform:** EBSCOhost
**Fields:** CINAHL subject headings, title, and abstract
**Limits applied in interface:** 1 January 2010–24 June 2026; no language limit

(

(MH "Triage+")

OR TI (

triage OR triaging OR "emergency department triage"

OR "emergency triage" OR "urgent care triage"

OR "acute care triage" OR "telephone triage"

OR teletriage OR "tele-triage" OR "video triage"

OR "remote triage" OR "virtual triage" OR "digital triage"

OR "electronic triage" OR "e-triage"

OR "patient prioritization" OR "patient prioritisation"

OR "clinical prioritization" OR "clinical prioritisation"

OR "virtual front door" OR "digital front door"

)

OR AB (

triage OR triaging OR "emergency department triage"

OR "emergency triage" OR "urgent care triage"

OR "acute care triage" OR "telephone triage"

OR teletriage OR "tele-triage" OR "video triage"

OR "remote triage" OR "virtual triage" OR "digital triage"

OR "electronic triage" OR "e-triage"

OR "patient prioritization" OR "patient prioritisation"

OR "clinical prioritization" OR "clinical prioritisation"

OR "virtual front door" OR "digital front door"

)

)

AND

(

(MH "Artificial Intelligence+")

OR (MH "Machine Learning+")

OR (MH "Natural Language Processing+")

OR (MH "Telehealth+")

OR (MH "Decision Support Systems, Clinical+")

OR TI (

"artificial intelligence" OR "machine learning" OR "deep learning"

OR neural network* OR random forest* OR gradient boost*

OR XGBoost OR support vector machine* OR transformer*

OR large language model* OR "natural language processing"

OR NLP OR predictive model* OR prediction model*

OR "risk prediction" OR "clinical decision support"

OR decision support system* OR computerized OR computerised

OR automated OR automation OR telehealth OR telemedicine

OR "remote consultation" OR "remote assessment"

OR "digital health" OR ehealth OR "e-health"

OR mhealth OR "m-health" OR symptom checker*

OR symptom assessment tool*

)

OR AB (

"artificial intelligence" OR "machine learning" OR "deep learning"

OR neural network* OR random forest* OR gradient boost*

OR XGBoost OR support vector machine* OR transformer*

OR large language model* OR "natural language processing"

OR NLP OR predictive model* OR prediction model*

OR "risk prediction" OR "clinical decision support"

OR decision support system* OR computerized OR computerised

OR automated OR automation OR telehealth OR telemedicine

OR "remote consultation" OR "remote assessment"

OR "digital health" OR ehealth OR "e-health"

OR mhealth OR "m-health" OR symptom checker*

OR symptom assessment tool*

)

)

Apply the publication-date limiter in the EBSCOhost interface and record the exact date and retrieval count.

**3. Web of Science Core Collection**

**Platform:** Clarivate Web of Science
**Field:** Topic, covering title, abstract, author keywords, and Keywords Plus
**Indexes searched:** List the exact indexes selected, such as SCI-EXPANDED, SSCI, ESCI, and CPCI
**Timespan:** 2010–2026

TS=(

(

triage OR triaging OR "emergency department triage"

OR "emergency triage" OR "urgent care triage"

OR "acute care triage" OR "telephone triage"

OR teletriage OR "tele-triage" OR "video triage"

OR "remote triage" OR "virtual triage" OR "digital triage"

OR "electronic triage" OR "e-triage"

OR "patient prioritization" OR "patient prioritisation"

OR "clinical prioritization" OR "clinical prioritisation"

OR "virtual front door" OR "digital front door"

)

AND

(

"artificial intelligence" OR "machine learning" OR "deep learning"

OR "neural network*" OR "random forest*" OR "gradient boost*"

OR XGBoost OR "support vector machine*" OR transformer*

OR "large language model*" OR "natural language processing"

OR NLP OR "predictive model*" OR "prediction model*"

OR "risk prediction" OR "clinical decision support"

OR "decision support system*" OR computerized OR computerised

OR automated OR automation OR telehealth OR telemedicine

OR "remote consultation" OR "remote assessment"

OR "digital health" OR ehealth OR "e-health"

OR mhealth OR "m-health" OR "symptom checker*"

OR "symptom assessment tool*"

)

)

AND PY=(2010-2026)

Record the exact Web of Science indexes selected.

**4. Scopus**

**Platform:** Elsevier Scopus
**Fields:** Title, abstract, and keywords
**Publication years:** 2010–2026

TITLE-ABS-KEY(

(

triage OR triaging OR "emergency department triage"

OR "emergency triage" OR "urgent care triage"

OR "acute care triage" OR "telephone triage"

OR teletriage OR "tele-triage" OR "video triage"

OR "remote triage" OR "virtual triage" OR "digital triage"

OR "electronic triage" OR "e-triage"

OR "patient prioritization" OR "patient prioritisation"

OR "clinical prioritization" OR "clinical prioritisation"

OR "virtual front door" OR "digital front door"

)

AND

(

"artificial intelligence" OR "machine learning" OR "deep learning"

OR "neural network*" OR "random forest*" OR "gradient boost*"

OR XGBoost OR "support vector machine*" OR transformer*

OR "large language model*" OR "natural language processing"

OR NLP OR "predictive model*" OR "prediction model*"

OR "risk prediction" OR "clinical decision support"

OR "decision support system*" OR computerized OR computerised

OR automated OR automation OR telehealth OR telemedicine

OR "remote consultation" OR "remote assessment"

OR "digital health" OR ehealth OR "e-health"

OR mhealth OR "m-health" OR "symptom checker*"

OR "symptom assessment tool*"

)

)

AND PUBYEAR > 2009

AND PUBYEAR < 2027
