# Supplementary material for: Technological Integration in Triage With AI and Machine Learning Compared With Conventional Triage and Narrative Synthesis of Telehealth for Patient Prioritization in Emergency and Acute Care Settings: Systematic Review and Meta-Analysis
Source: J Med Internet Res. 2026 Oct 19;28:e93931. doi: 10.2196/93931 (PMC13679870; doi:10.2196/93931)
Supplement: Supplementary file 3 [file jmir-v28-e93931-s002.docx]

**Supplement Table S1A - Meta-analysis-eligible included studies (n = 13)**

| Study | Setting & N | Design | Intervention/model | Predictors/ inputs | Comparator | Outcome(s) | Key metrics (extractable) | Thresholding | Calibration | Subgroups | Implementation/ workflow |
| --- | --- | --- | --- | --- | --- | --- | --- | --- | --- | --- | --- |
| Määttä et al (2023) [78] | Single university hospital ED; adults (≥17y) triaged; n=248[55] | Prospective/ real-world evaluation of CDSS at triage; clinician survey | CDSS generating probabilities of relevant conditions +urgency estimate | Patient-entered/triage-entered symptoms/history; algorithm outputs risk % and urgency | Triage nurses’ urgency assessment / standard triage | Agreement/accuracy of urgency classification; usability (SUS) | Exact-match accuracy 45.5% (97/213); “critical” sensitivity~70% (7/10) | Risk threshold 15% used to classify relevant conditions | Not reported | Not reported | Used in real triage; SUS mean68.0 (SD 13.2) |
| Yun et al (2021) [69] | Single ED; adults; n=80,433 [47] | Retrospective cohort model evaluation using initial triage+ EHR variables | XGBoost and DNN for critical care prediction | KTAS + age/sex + arrival mode+ vitals + mental status + chief complaint + history | Baseline logistic model using KTAS | Need for critical care | AUROC: XGB 0.861; DNN 0.833; baseline 0.796; NRI reported | ROC-based; no single clinical cut point emphasized in abstract | HL P>.05 for XGB; HL P<.001 for DNN; calibration plot | Not clearly reported in abstract | Explainability (partial dependence); no prospective workflow evaluation |
| Horng et al (2017) [18] | Single academic ED (BIDMC); n=230,936 visits[54] | Retrospective cohort; EHR +free-text at triage | ML/NLP automated trigger for sepsis decision support | Demographics/vitals; chief complaint; free-text triage note (bag-of-words/topic models) | Models without text (vitals-only) and with structured chief complaint | Diagnosed infection (proxy for screening trigger) | Test AUC: vitals-only 0.67; chief-complaint 0.83; bag-of-words 0.86; topic 0.85 | Not specified (trigger operating point not in abstract) | Not reported | Not reported | Retrospective derivation/validation; framed as trigger for CDS |
| Levin et al (2017) [70] | 2 EDs (academic + community); adults; n=172,726 (2013-2015)[73] | Retrospective cohort; e-triage run in parallel to usual triage | “E-triage” ML risk stratification mapped to triage levels | Triage + EHR history; structured data + model-described feature sets | Emergency Severity Index (ESI) | Critical care, emergency procedure; hospitalization; troponin/lactate | AUC range 0.73-0.92 (by outcome/site); ESI-3 reclassification reported | Risk thresholds map probabilities to triage levels (details in figures) | Not clearly reported | ESI level 3 focus; site comparisons | Parallel evaluation; highlights risk-based differentiation within ESI-3 |
| Goto et al (2019) [79] | NHAMCS paediatric ED visits; n=52,037 (2007-2015)[51] | Prognostic model study (TRIPOD); national survey dataset | Lasso, RF, gradient boosting, DNN | Routine triage data (demographics, vitals, chief complaint, etc.) | Logistic regression using 5-levelconventional triage classification | Critical care; hospitalization | Critical care C-stat 0.85 vs 0.78 (ref); hospitalization 0.80 vs 0.73 (ref) | Decision curve analysis across threshold probabilities | Not reported | Not reported | No prospective deployment; includes decision curves |
| Hong et al (2018) [81] | 1 academic + 2 community EDs; adults; n=560,486 (2014-2017)[53] | Retrospective cohort; 972 vars/visit | Admission prediction (LR, XGB, DNN): triage-only vs history-only vs full | Triage-only; history-only; full feature set; also, low-dimensional XGB | Across-feature-set comparisons (triage vs history vs full) | Hospital admission vs discharge | Test AUC: triage-only ~0.87; full set: LR 0.91, XGB 0.92, DNN 0.92 | ROC-based; no single threshold highlighted | Not reported in abstract | Not reported in abstract | No prospective impact evaluation; notes max performance at ≤50% training size |
| Raita et al (2019) [20] | NHAMCS adult ED visits; ≥18y; n=135,470 (2007-2015)[48] | Prognostic model study; national dataset | Lasso, RF, GBDT, DNN | Demographics, vitals, chief complaint, comorbidities (triage-available) | ESI-based logistic regression (ESI levels) | Critical care; hospitalization | Critical care AUC 0.86 vs 0.74 (ref); hospitalization AUC 0.82 vs 0.69 (ref) | Decision curves across threshold probs | Not reported | ESI-level under/over-triage patterns | Nationally representative; focuses on reducing under-/over-triage |
| Hinson et al (2025) [77] | 3 EDs; adults; n=174,648(pre=83,404; post= 91,244)[84] | Before-after implementation evaluation | EHR-integrated ML triage tool (Triage GO) | EHR variables available at triage (exact list not in abstract) | Standard clinician triage (pre) | High-severity designation vs critical illness | Sensitivity 78.9%→83.1%; specificity 74.3%→76.8% | Tool output mapped to high-severity triage category | Not reported | Stratified analyses described by race, ethnicity, language, sex | Production tool at triage; evaluates classification performance changes |
| Rendell et al [71] | NSW ED network; adults >16; n=1,721,294 (2014)[85] | Retrospective ML development/validation | START2 admission prediction (best 1-NNwith information gain features) | Triage category, age, ambulance arrival, prior inpatient/ED use, time/day, presenting problem | Original START model (LR) | Hospital admission | AUC up to 0.8267; accuracy ~74.8-75.2% | Not specified | Not reported | Presenting problem stratification | Designed for CDS/flow; no prospective deployment |
| Kwon et al (2018) [75] | National ED dataset (151 EDs) + external hospital; n=11,656,559[49] | Retrospective derivation/validation + external validation | Deep-learning Triage and Acuity Score (DTAS) | Age/sex, chief complaint, onset-to-arrival, arrival mode, trauma, initial vitals, mental status | KTAS; MEWS; LR; RF | Mortality; critical care; hospitalization | Mortality AUROC 0.935 (DTAS) vs 0.785 (KTAS); critical care AUROC 0.890; hospitalization AUROC 0.871 | Not specified | Not reported in abstract | External validation at separate hospital | Large-scale validation; designed for real-time use |
| Hwang and Lee [80] | National ED dataset; children <15; n=2,621,710 (2016-2017)[52] | Cross-sectional observational; RF trained on under sampled set | Random forest triage model | Structured triage data (NEDIS) | Conventional paediatric triage (paediatric KTAS) | Critical illness; hospitalization | AUROC 0.991 (critical illness), 0.943 (hospitalization) | Not specified | Not reported | Not reported | Population-scale performance; no prospective deployment |
| Yu et al (2020) [76] | Single tertiary ED; adults ≥18; n=86,304 (2016-2017)[50] | Retrospective model dev + internal validation | ML + DL using Initial Nursing Assessment (INA) | INA: demographics, vitals, ESI severity index, chief complaint; low-dimensional 4-variable model | KTAS model; SOFA | ICU admission or ED death | AUC: INA LR 0.872; INA DL 0.876; KTAS 0.768; SOFA 0.740 | ROC-based; no single operating point highlighted | Not reported | Severity strata described (KTAS strata) | Not prospectively deployed; INA designed for efficient triage |
| Brann et al (2024) [19] | 4 EDs; adults; n=1,059,386 final cohort[86] | Retrospective cohort; train n=950,921; test n=108,465 | NLP + ensemble to predict sepsis at triage (time-of-triage model) | Nursing triage notes (NLP + reduction) + triage-time clinical data | No triage-scale comparator in abstract | Sepsis (infection + organ dysfunction) | AUC 0.94; Se 0.87; Sp 0.85; PPV 0.18; NPV 0.99 | FPR fixed at 0.15 | Not reported | Stratified by hospital; alternate CDC definition tested | Retrospective; targets missed screening at triage |

Footnote: ED, Emergency Department; CDSS, Clinical Decision Support System; SUS, System Usability Scale; AUROC/AUC, Area Under the Receiver Operating Characteristic Curve; ML, Machine Learning; NLP, Natural Language Processing; EHR, Electronic Health Record; KTAS, Korean Triage and Acuity Scale; ESI, Emergency Severity Index; MEWS, Modified Early Warning Score; LR, Logistic Regression; RF, Random Forest; GBDT, Gradient-Boosted Decision Trees; DNN, Deep Neural Network; XGB, Extreme Gradient Boosting; DTAS, Deep-learning Triage and Acuity Score; INA, Initial Nursing Assessment; CDS, Clinical Decision Support; ICU, Intensive Care Unit; HL, Hosmer–Lemeshow goodness-of-fit test; NRI, Net Reclassification Improvement; PPV, Positive Predictive Value; NPV, Negative Predictive Value; Se, Sensitivity; Sp, Specificity; FPR, False Positive Rate; NHAMCS, National Hospital Ambulatory Medical Care Survey; NEDIS, National Emergency Department Information System; CDC, Centers for Disease Control and Prevention.

References:

18. Horng S, Sontag DA, Halpern Y, Jernite Y, Shapiro NI, Nathanson LA. Creating an automated trigger for sepsis clinical decision support at emergency department triage using machine learning. PLoS One. 2017;12(4):e0174708. [doi: ] [Medline: 28384212]

19. Brann F, Sterling NW, Frisch SO, Schrager JD. Sepsis prediction at emergency department triage using natural language processing: retrospective cohort study. JMIR AI. Jan 25, 2024;3(1):e49784. [doi: ] [Medline: 38875594]

20. Raita Y, Goto T, Faridi MK, Brown DFM, Camargo CA, Hasegawa K. Emergency department triage prediction of clinical outcomes using machine learning models. Crit Care. Feb 22, 2019;23(1):64. [doi: ] [Medline: 30795786]

69. Yun H, Choi J, Park JH. Prediction of critical care outcome for adult patients presenting to emergency department using initial triage information: an XGBoost wlgorithm analysis. JMIR Med Inform. Sep 20, 2021;9(9):e30770. [doi: ] [Medline: 34346889]

70. Levin S, Toerper M, Hamrock E, et al. Machine-learning-based electronic triage more accurately differentiates patients with respect to clinical outcomes compared with the emergency severity index. Ann Emerg Med. May 2018;71(5):565-574. [doi: ] [Medline: 28888332]

71. Rendell K, Koprinska I, Kyme A, Ebker-White AA, Dinh MM. The Sydney Triage to Admission Risk Tool (START2) using machine learning techniques to support disposition decision-making. Emerg Med Australas. Jun 2019;31(3):429-435. [doi: ] [Medline: 30469164]

75. Kwon J myoung, Lee Y, Lee Y, Lee S, Park H, Park J. Validation of deep-learning-based triage and acuity score using a large national dataset. PLoS One. 2018;13(10):e0205836. [doi: ] [Medline: 29793315]

76. Yu JY, Jeong GY, Jeong OS, Chang DK, Cha WC. Machine learning and initial nursing assessment-based triage system for emergency department. Healthc Inform Res. Jan 2020;26(1):13-19. [doi: ] [Medline: 32082696]

77. Hinson JS, Levin SR, Steinhart BD, et al. Enhancing emergency department triage equity with artificial intelligence: outcomes from a multisite implementation. Ann Emerg Med. Mar 2025;85(3):288-290. [doi: ] [Medline: 39570253]

78. Määttä J, Lindell R, Hayward N, et al. Diagnostic performance, triage safety, and usability of a clinical decision support system within a university hospital emergency department: algorithm performance and usability study. JMIR Med Inform. Aug 31, 2023;11(1):e46760. [doi: ] [Medline: 37656018]

79. Goto T, Camargo CA Jr, Faridi MK, Freishtat RJ, Hasegawa K. Machine learning-based prediction of clinical outcomes for children during emergency department triage. JAMA Netw Open. Jan 4, 2019;2(1):e186937. [doi: ] [Medline: 30646206]

80. Hwang S, Lee B. Machine learning-based prediction of critical illness in children visiting the emergency department. PLoS One. 2022;17(2):e0264184. [doi: ] [Medline: 35176113]

81. Hong WS, Haimovich AD, Taylor RA. Predicting hospital admission at emergency department triage using machine learning. PLoS One. 2018;13(7):e0201016. [doi: ] [Medline: 30028888]
