# Supplementary material for: Technological Integration in Triage With AI and Machine Learning Compared With Conventional Triage and Narrative Synthesis of Telehealth for Patient Prioritization in Emergency and Acute Care Settings: Systematic Review and Meta-Analysis
Source: J Med Internet Res. 2026 Oct 19;28:e93931. doi: 10.2196/93931 (PMC13679870; doi:10.2196/93931)
Supplement: Supplementary file 4 [file jmir-v28-e93931-s003.docx]

**Supplement Table S1B - Narrative-only included studies (n = 14)**

| **Study** | **Setting & N** | **Design** | **Intervention/technology** | **Inputs** | **Comparator** | **Outcome(s)** | **Key results** | **Implementation/ workflow** |
| --- | --- | --- | --- | --- | --- | --- | --- | --- |
| Çetin et al (2023) [89] | ED triage; adults; Pré n=16,409; post n=7,765[66] | Quasi-experimental pre/post | Digital triage DSS aligned with ESI v4 + ATS | Nurse-entered triage data | Usual nurse triage | Triage accuracy; triage time; satisfaction | Accuracy 96.9%→99.1%; time 3.72→2.72 min; satisfaction 4.52/5 | Point-of-care tool used by triage nurses; no AUC |
| Cho et al (2022) [88] | ED triage unit; 19 nurses;1,063 tasks[65] | Prospective interventional workflow comparison | RMIS-AI voice-based AI documentation | Speech-to-text + NLP | Manual documentation | Task time; completion accuracy | Per-task time reduced ~39-53%; accuracy improved | Documentation/efficiency tool; not acuity prediction |
| Joshi et al (2020) [82] | Urban community ED; Pré n=2,225; post n=2,678[59] | Before-after | ED tele-intake at first contact | Telehealth intake process | Usual intake/triage | LWBS; door-to-provider; LOS | LWBS 2.6%→1.2%; door-to-provider 41→25 min; LOS (discharged) 3.3→3.5 h | Operational/ throughput-focused |
| Rademacher et al (2019) [83] | ED; tele-screened n=2,532 vs controls n=39,370[62] | Matched cohort | Telehealth screening in triage | Telehealth consult | Standard triage pathway | LOS; LWBS; admission | LOS 4.0 vs 5.8 h; LWBS 0.6% vs 3.8%; admission 18.7% vs 28.0% | Operational impact; not ML |
| Izzo et al (2018) [84] | ED; pre n=7,326 vs intervention n=6,586[58] | Before-after | Tele-triage / telemedicine intake | Video/remote triage | Standard triage | Throughput/staffing/exposure metrics | Reported in paper (throughput + operational endpoints) | COVID-era tele-triage deployment |
| Cotte et al (2022) [74] | ED patients; n=378[63] | Prospective comparison | Ada symptom checker urgency advice | Patient-entered symptoms | Manchester Triage System (MTS) | Agreement; over-/under-triage | Over-triage 49.2%; under-triage 20.6%; major under-triage 5.6% | Patient-facing front-door tool |
| Meer et al (2024) [87] | ED walk-ins; n=2,500[64] | Prospective observational validation | Symptom checker triage urgency | Patient-entered symptoms | MTS | Agreement; safety | Agreement 54%; under triage 13.3%; over triage 47.0%; Se 69.9%, Sp 56.7% (urgent vs non-urgent) | Patient-facing tool tested in real ED |
| Liu et al (2024) [90] | Adult ED presentations; n=877[67] | Retrospective chart review validation | ED Triage App | Patient-entered symptoms + demographics | CTAS nurse assignment | Agreement; sensitivity/specificity | High-acuity (CTAS 1-2): Se 82.6%, Sp 68.3%; urgent (CTAS 1-3): Se 85.0%, Sp 62.6% | Digital intake supporting front-door triage |
| Gren et al (2023) [85] | Paediatrics; n=801 (video 371; telephone 383)[57] | RCT | Video triage | Video consult | Telephone triage | ED visit 48h; hospitalization; satisfaction | ED visit 48h: 3.2% vs 3.5%; hospitalization 0.3% vs 0.5% | Tele-triage modality trial |
| Campbell et al (2014) [86] | Primary care same-day requests; n=20,990[56] | Cluster RCT | GP-/nurse-led telephone triage | Telephone triage protocols | Usual care access | Service use; safety; costs | Increased contacts; detailed endpoints in paper | Non-AI tele-triage at access point |
| Lin et al (2020) [72] | ED; n=4,512[61] | Retrospective cohort comparison | Telemedicine “double triage” | Telemedicine screening workflow | Traditional triage | Waiting time; LOS; 72h return; mortality | Waiting 15.0 vs 17.6 min; LOS 188.0 vs 220.3 min; 72h return similar | Pandemic workflow redesign |
| Inokuchi et al (2022) [73] | After-hours house-call telephone triage service; adults ≥16 y; n≈19,114 | Retrospective cohort; ML model development and validation | ML models for predicting undertriage | Telephone triage level; age; sex; chief complaint categories; comorbidities | Standard telephone triage | Undertriage | Best performance with RF and XGB (AUROC ≈0.80-0.81); ML outperformed simpler models | Decision-support for telephone triage |
| Fernandes et al (2020) [92] | ED datasets: Portugal n=599,276; USA n=267,257[68] | Retrospective ML+NLP dev/validation | ML + NLP for ICU admission prediction | Vitals, pain, demographics + free text | Triage priority reference (MTS/ESI) | ICU admission prediction | AUROC reported in figures/tables (not cleanly extractable from text layer) | Cross-setting model validation; not prospectively deployed |
| Liu et al (2021) [91] | Single tertiary ED; retrospective n=22,272 + prospective cohort (size not stated in extractable text)[69] | Retrospective + prospective evaluation | ML system with explanation for critical-care need | Age/sex/arrival mode/time; vitals; consciousness; chief complaint; blood sugar; pain; ETS | Traditional electronic triage system | Critical-care need; mis-triage | Retrospective AUC 0.875 vs 0.843; prospective mis-triage 1.2%→0.9% | Includes prospective component + explainability |

Footnote: ED, Emergency Department; DSS, Decision Support System; ESI, Emergency Severity Index; ATS, Australasian Triage Scale; AUC/AUROC, Area Under the Receiver Operating Characteristic Curve; ML, Machine Learning; NLP, Natural Language Processing; AI, Artificial Intelligence; LOS, Length of Stay; LWBS, Left Without Being Seen; GP, General Practitioner; MTS, Manchester Triage System; CTAS, Canadian Triage and Acuity Scale; ICU, Intensive Care Unit; RCT, Randomized Controlled Trial; Se, Sensitivity; Sp, Specificity; RF, Random Forest; XGB, Extreme Gradient Boosting; ETS, Electronic Triage System.

References:

72. Lin CH, Tseng WP, Wu JL, et al. A double triage and telemedicine protocol to optimize infection control in an emergency department in Taiwan during the COVID-19 pandemic: retrospective feasibility study. J Med Internet Res. Jun 23, 2020;22(6):e20586. [doi: ] [Medline: 32544072]

73. Inokuchi R, Iwagami M, Sun Y, Sakamoto A, Tamiya N. Machine learning models predicting undertriage in telephone triage. Ann Med. Dec 2022;54(1):2990-2997. [doi: ] [Medline: 36286496]

74. Cotte F, Mueller T, Gilbert S, et al. Safety of triage self-assessment using a symptom assessment app for walk-in patients in the emergency care setting: observational prospective cross-sectional study. JMIR Mhealth Uhealth. Mar 28, 2022;10(3):e32340. [doi: ] [Medline: 35343909]

82. Joshi AU, Randolph FT, Chang AM, et al. Impact of emergency department tele-intake on left without being seen and throughput metrics. Acad Emerg Med. Feb 2020;27(2):139-147. [doi: ] [Medline: 31733003]

83. Rademacher NJ, Cole G, Psoter KJ, et al. Use of telemedicine to screen patients in the emergency department: matched cohort study evaluating efficiency and patient safety of telemedicine. JMIR Med Inform. May 8, 2019;7(2):e11233. [doi: ] [Medline: 31066698]

84. Izzo JA, Watson J, Bhat R, et al. Diagnostic accuracy of a rapid telemedicine encounter in the emergency department. Am J Emerg Med. Nov 2018;36(11):2061-2063. [doi: ] [Medline: 30209006]

85. Gren C, Hasselager AB, Linderoth G, et al. Video triage in calls concerning children with fever at an out-of-hours medical helpline: a prospective quality improvement study. Scand J Trauma Resusc Emerg Med. Aug 29, 2023;31(1):41. [doi: ] [Medline: 37644510]

86. Campbell JL, Fletcher E, Britten N, et al. Telephone triage for management of same-day consultation requests in general practice (the ESTEEM trial): a cluster-randomised controlled trial and cost-consequence analysis. The Lancet. Nov 2014;384(9957):1859-1868. [doi: ]

87. Meer A, Rahm P, Schwendinger M, et al. A symptom-checker for adult patients visiting an interdisciplinary emergency care center and the safety of patient self-triage: real-life prospective evaluation. J Med Internet Res. Jun 27, 2024;26:e58157. [doi: ] [Medline: 38809606]

88. Cho A, Min IK, Hong S, Chung HS, Lee HS, Kim JH. Effect of applying a real-time medical record input assistance system with voice artificial intelligence on triage task performance in the emergency department: prospective interventional study. JMIR Med Inform. Aug 31, 2022;10(8):e39892. [doi: ] [Medline: 36044254]

89. Çetin SB, Cebeci F, Eray O. The effect of computer-based decision support system on emergency department triage: non-randomised controlled trial. Int Emerg Nurs. Sep 2023;70:101341. [doi: ] [Medline: 37708790]

90. Liu V, Kaila M, Koskela T. Triage accuracy and the safety of user-initiated symptom assessment with an electronic symptom checker in a real-life setting: instrument validation study. JMIR Hum Factors. Sep 26, 2024;11:e55099. [doi: ] [Medline: 39326038]

91. Liu Y, Gao J, Liu J, et al. Development and validation of a practical machine-learning triage algorithm for the detection of patients in need of critical care in the emergency department. Sci Rep. 2021;11(1):24044. [doi: ]

92. Fernandes M, Mendes R, Vieira SM, et al. Predicting intensive care unit admission among patients presenting to the emergency department using machine learning and natural language processing. PLoS One. 2020;15(3):e0229331. [doi: ] [Medline: 32126097]
